# Volatile Composition of Brazilian Stingless Bee Propolis

**DOI:** 10.3390/molecules31132363

**Published:** 2026-07-05

**Authors:** Mariana Budóia Gabriel, Guilherme Perez Pinheiro, Leandro Wang Hantao, Alexandra Christine Helena Frankland Sawaya

**Affiliations:** 1 Laboratory of Metabolomics and Mass Spectrometry (LabMetaMass), Faculty of Pharmaceutical Science, Universidade Estadual de Campinas (UNICAMP), Campinas 13083-871, SP, Brazil; marianabudoia@gmail.com (M.B.G.); pinheiro.gperez@gmail.com (G.P.P.); 2 Department of Analytical Chemistry, Institute of Chemistry, Universidade Estadual de Campinas (UNICAMP), Campinas 13083-862, SP, Brazil; wang@unicamp.br

**Keywords:** geopropolis, volatile compounds, stingless bees, GC-MS, untargeted metabolomics

## Abstract

Stingless bees, or meliponines, are essential pollinators in Brazil, with over 300 described species. These bees produce propolis or geopropolis (characterized by the incorporation of mineral material and clay) used to protect their nests. This product has aroused increasing scientific interest due to its therapeutic potential, including antimicrobial and anti-inflammatory activities. However, there is still limited knowledge about its volatile composition, which can vary according to the bee species and the botanical origin of the resins, influencing their biological and aromatic properties. The purpose of this study was to characterize the volatile composition of (geo)propolis produced by different species of native stingless bees from the Southeastern region of Brazil (São Paulo and Minas Gerais) and to detect if this composition is influenced by the species or by the region. The samples were analyzed by headspace solid-phase microextraction (HS-SPME) coupled to gas chromatography–mass spectrometry (GC-MS). The results indicated that, despite some variations, the chemical profile for each species was mostly constant between regions. In São Paulo, about 25% of the features varied between species, whereas in Minas Gerais, only 5% showed significant differences, although one species (*Melipona quadrifasciata*) presented a very constant composition. Although the local vegetation determines the supply of resins for these bees, differences in the chemical composition of propolis are a result of a species’ choice of plant species.

## 1. Introduction

Stingless bees, also known as meliponines or native bees, play an essential role in the pollination of Brazilian flora. In Brazil, more than 300 species have been recorded, although this number may increase with the description of new species by science. These insects generally build their nests in natural cavities in tree trunks [1]. To protect the colony, they collect plant resins that are later modified with secretions such as wax and saliva, resulting in the formation of propolis. This material is used in the internal lining of the nest and in defense against invaders, and can even promote their immobilization and mummification [2]. Although some species of stingless bees produce geopropolis, a mixture of plant resins, wax and soil, differing from propolis due to its mineral content [3], both are called propolis in this study, for the sake of simplicity.

In recent years, scientific interest in stingless bee propolis has increased due to its biological potential. Studies report antimicrobial, anti-inflammatory, antioxidant, and antiviral activities associated with different species of meliponines [4]. In this context, it becomes relevant to understand possible variations in its chemical composition, aiming to correlate the composition to the observed biological activities.

However, most research focuses on ethanolic or hydroethanolic extracts, while the volatile fraction remains mostly unexplored. A recent review found only 12 studies regarding volatile compounds present in propolis and geopropolis of Brazilian stingless bees [5]. Furthermore, several studies have limitations regarding the precise identification of the bee species that produced the propolis, as well as the standardization of extraction methods. The volatile compounds of propolis represent a chemically diverse fraction, with potential contributions to its biological properties and aromatic characteristics. These profiles can vary according to geographic origin, available flora, and the bee species involved in collection. Among the main compounds which were previously described are monoterpenes and sesquiterpenes such as tricyclene, α-pinene, camphene, β-pinene, ρ-cymene, limonene, terpinen-4-ol, α-terpineol, myrtenol, α-copaene, β-caryophyllene, γ-murolene, δ-cadinene and guaiol [6,7,8,9].

In previous studies, we reviewed the known volatile composition of Brazilian stingless bee propolis [5] as well as standardized a method for the extraction and analysis of these molecules [10]. Given this scenario, we hypothesized that, by applying this method to compare stingless bee propolis from different bee species and regions, we could determine whether the volatile chemical composition of (geo)propolis could be influenced by the bee species and/or the geographical origin of the samples.

## 2. Results

### 2.1. Identification of the Volatile Compounds

From the 227 features detected by MS-Dial, it was possible to identify 76 substances based on their fragmentation spectra and retention indexes. The identified compounds presented in Table 1 are organized according to the total features. Supplementary Table S1 presents all identified and unidentified features detected. Their identification was based on the calculated retention index (RI), the theoretical retention index (AI), comparison with the NIST library and MS-Dial proposal, and the average percentage of the compounds identified in the samples. It should be noted that the retention times (RT) reported by MS-DIAL correspond to average values for each feature obtained after feature alignment between all analyzed samples. Due to the complexity of the matrix and the small chromatographic variations inherent in the analyses, the reported RTs represent aligned average values and not individual measurements. Consequently, small differences between the experimental retention indices (RI), calculated from a homologous series of alkanes, and those described in the literature may be observed. Therefore, the identification of the compounds was based on the combination of retention indices and the comparison of mass spectra with reference libraries.

### 2.2. Analytical Reproducibility

Principal component analysis (PCA), shown in Figure S1 (Supplementary), highlights the clustering of quality control samples (QCs), indicating good reproducibility of the analytical method. On the PC1 axis, the QCs are concentrated close to zero. Although the QCs are not completely centered along PC2, their compact distribution indicates good analytical stability and reproducibility of the method. The slight shift observed may be related to the high biological heterogeneity of the sample set, since the QC pool was composed of samples from different bee species, represented in non-uniform proportions.

### 2.3. Comparison of Propolis Samples from the Same Bee Species from Different States

Samples from the Southeast region were obtained in two states: São Paulo (Jaguariúna) and Minas Gerais (Muzambinho and Cabo Verde). From this region of the country, 60 samples of (geo)propolis from eight different bee species were obtained. Initially, the samples were analyzed by species, with the aim of identifying possible variations between the states of SP and MG for each species (*Plebeia droryana*, *Tetragonisca angustula*, *Nannotrigona testaceicornis* and *Melipona quadrifasciata*). Subsequently, the samples from each state were grouped, and the different species within each state (SP and MG) were compared. Initially, the GC-MS profiles of the four species with the highest number of available samples and that were present in all three study locations, Cabo Verde and Muzambinho (Minas Gerais State) and Jaguariúna (São Paulo State), were evaluated.

#### 2.3.1. *Melipona quadrifasciata*

**Figure 1:**
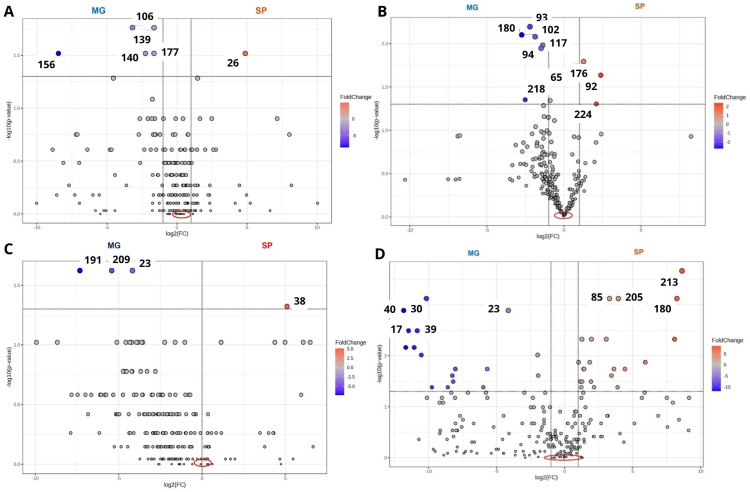
*Volcano plots* of the samples of (**A**) *Plebeia droryana* from MG and SP; (**B**) *Tetragonisca angustula* from MG and SP; (**C**) *N. testaceicornis* from MG and SP; (**D**) *M. quadrifasciata* from MG and SP. MG—Minha Gerais/SP—São Paulo. The number of samples and names are described in Table 2. Red circles indicate the least variable features.

Eighteen samples of *Melipona quadrifasciata* propolis were analyzed, including 12 samples from the state of Minas Gerais (10—Muzambinho and 2—Cabo Verde) and six samples from the state of São Paulo (Jaguariúna). The *volcano plot* (Figure 1D) analysis presented 22 features that were significantly (*p* < 0.05) more abundant in São Paulo, shown in red: wherein features 85, 180, 205, and 213 stood out, indicating the greatest differences between the groups. From these, α-alaskene (feature 205) and trans-cadina-1,4-diene (feature 213) were identified. On the other hand, 17 features, shown in blue, presented significantly greater abundance in samples from Minas Gerais, notably features 17, 23, 30, 39, and 40; feature 40 was identified as sabinene.

**Table 2:** Identification of the samples used in the study (code name, species, common name, site of collection and number of samples).

Identification	Species	Common Name	Site of Collection	Total Number of Samples
Fv1J—Fv2J	*Frieseomelitta varia*	*Marmelada*	Jaguariúna—SP	02
Mb1J—Mb5J	*Melipona bicolor*	*Guarupu*	Jaguariúna—SP	05
Mm1J—Mm4J	*Melipona marginata*	*Manduri*	Jaguariúna—SP	04
Mq1J—Mq6J	*Melipona quadrifasciata*	*Mandaçaia*	Jaguariúna—SP	06
Mq1M—Mq10M	*Melipona quadrifasciata*	*Mandaçaia*	Muzambinho—MG	10
Mq1CV—Mq2CV	*Melipona quadrifasciata*	*Mandaçaia*	Cabo Verde—MG	02
Mr1CV—Mr5CV	*Melipona rufiventris*	*Uruçu*	Cabo Verde—MG	05
Nt1J—Nt3J	*Nannotrigona testaceicornis*	*Iraí*	Jaguariúna—SP	03
Nt1M—Nt6M	*Nannotrigona testaceicornis*	*Iraí*	Muzambinho—MG	06
Pd1J—Pd5J	*Plebeia droryana*	*Mirim droriana*	Jaguariúna—SP	05
Pd1CV—Pd6CV	*Plebeia droryana*	*Mirim droriana*	Cabo Verde—MG	06
Ta1J—Ta3J	*Tetragonisca angustula*	*Jataí*	Jaguariúna—SP	03
Ta1M—Ta3M	*Tetragonisca angustula*	*Jataí*	Muzambinho—MG	03

A total of 39 features were significantly different between the regions, corresponding to 17% of the total features analyzed, while 188 were not significantly different, shown in gray in Figure 1D. The features positioned closest to zero on both axes (circled in red) varied the least between the samples. For example, compounds 2-epi-α-funebrene (feature 140), trans-caryophyllene (feature 161), β-selinene (feature 179) and muurola-4(14),5-diene (feature 187) were identified as part of this group.

The PCA (Figure S2A) describes only 25.8% of the variability between samples and most features showed no significant differences between the *Melipona quadrifasciata* samples from the two states (Figure 1D). Therefore, most of the volatile composition remained stable. However, some significant differences were identified. Figure S3 shows representative chromatograms of propolis samples from Minas Gerais and São Paulo. In these chromatograms, the features which were shown in red or blue in the volcano plot are marked. The variation in the chromatographic profiles was due to the relative intensity of the features, in addition to the absence of specific peaks in some samples (for example, Figure S3 Mq1J). Nevertheless, in general, the volatile composition was stable between the two states for this species.

#### 2.3.2. *Tetragonisca angustula*

Six samples of propolis produced by *Tetragonisca angustula* bees were analyzed, including three from Muzambinho (Minas Gerais) and three from Jaguariúna (São Paulo). Principal component analysis (PCA), presented in Figure S2B, showed that the PC1 axis explained 49.4% of the total variability of the data, indicating that the main variation occurred between sample Ta3M (MG) and the other samples. The PC2 axis explained 16.3% of the variability, further contributing to the separation of samples according to their region of origin. These results highlight some differences in the chemical profile of the propolis from the two states.

*Volcano plot* analysis (Figure 1B) indicates the compounds that vary significantly (*p* < 0.05) between groups. The three features shown in red presented significantly higher abundance in samples from São Paulo, corresponding to features 92, 176, and 224. Of these, para-ethylphenol (feature 92) and trans-veltonal (feature 224) were identified. On the other hand, seven features shown in blue presented higher relative abundance in samples from Minas Gerais, corresponding to features 65, 93, 94, 102, 117, 180, and 218. Of these, compounds para-cymene (feature 65), para-methylacetophenone (feature 94) and α-calamenene (feature 218) were identified.

Although 10 features presented significantly different intensities between samples from the two regions, the majority of 217 features did vary significantly, shown in gray in Figure 1B, which indicates a high degree of similarity between most of the samples. Additionally, the features positioned closer to the central axis of the graph, circled in red (features 13, 85, 129, 171, 205 and 230), varied the least. Feature 205 was identified as α-alaskene.

Figure S4 presents the chromatograms of samples from the states of São Paulo and Minas Gerais, marking the features that were significantly different between the groups in the volcano plot. Although the chromatographic profiles are similar, variation in the relative intensity of some features, as well as the absence of specific peaks, is observed.

Despite the slight variation in the volatile composition of propolis samples produced by *Tetragonisca angustula* bees from Minas Gerais and São Paulo, most of the detected features were not significantly different. Of the 227 features evaluated in this study, only 10 were significantly different, corresponding to approximately 4.4% of the total features detected. These results indicate that the similarity in the chemical composition predominates over the differences observed between samples from these regions.

#### 2.3.3. *Plebeia droryana*

Eleven samples of propolis produced by *Plebeia droryana* were analyzed, including six from the state of Minas Gerais (Cabo Verde) and five from the state of São Paulo (Jaguariúna). In the principal component analysis (PCA), presented in Figure S2C, principal component 1 (PC1) explained 49.3% of the total variability, indicating that the greatest difference observed occurred between sample Pd1J, from São Paulo, and the other samples. Principal component 2 (PC2), in turn, explained 16.8% of the variability, also contributing to the separation of samples according to their states of origin.

*Volcano plot* analysis (Figure 1A) indicates compounds that vary significantly (*p* < 0.05) between groups. Only feature 26 (shown in red) was significantly more abundant in samples from the state of São Paulo compared to those from Minas Gerais. Conversely, five features shown in blue were significantly more intense in samples from Minas Gerais. These were features 106, 139, 140, 156, and 177; of these, methyl thymol ether (feature 106) and 2-epi-α-funebrene (feature 140) were identified.

Although six features varied significantly between both regions, 221 features did not, and are shown in gray in Figure 1A. The features closest to zero, circled in red, corresponded to the smallest variations between the samples (39, 80, 98, 129, 163, 199 and 215). Of these, meta-cymenene (feature 80), myrtenal (feature 98), β-curcumene (feature 199) and α-cadinene (feature 215) were identified.

Figure S5 presents typical chromatograms of *Plebeia droryana* samples from São Paulo and Minas Gerais; the features that were significantly different between the groups are marked. When comparing the chromatograms, it can be observed that the Pd4J sample, from the São Paulo region, presented an intense peak related to feature 26 (Rt 4.51 min), while the other peaks remained close to the baseline. As the chromatograms were normalized by the peaks of greatest intensity, this apparent difference was accentuated. Although visual analysis indicates variations in peak intensities between the samples from São Paulo and Minas Gerais, statistical analyses showed that few differences were actually significant.

In general, the results show some significant differences in the chemical composition of *Plebeia droryana* propolis samples from the states of Minas Gerais and São Paulo; however, most of the features were not significantly different. Of the 227 features evaluated, only six were considered significantly different, corresponding to approximately 2.6% of the total analyzed. Thus, it is observed that the chemical similarity between the samples outweighs the differences. Furthermore, this constitutes the first study on the volatile compounds present in *Plebeia droryana* propolis.

#### 2.3.4. *Nannotrigona testaceicornis*

Nine samples of propolis produced by *Nannotrigona testaceicornis* were analyzed, including six from the state of Minas Gerais (Muzambinho) and three from the state of São Paulo (Jaguariúna). In the principal component analysis (PCA), presented in Figure S2D, the PC1 axis explained 64.2% of the total variability, indicating that the greatest variability occurred between sample Nt6M, from Minas Gerais, and the other samples. The PC2 axis explained 10% of the variability, mainly separating samples Nt1J, from the state of São Paulo, and Nt4M, from the state of Minas Gerais, from the other samples, although representing a smaller variability.

The volcano plot (Figure 1C) demonstrated that the compound, camphene (feature 38), shown in red, was significantly more intense (*p* < 0.05) in samples from the state of São Paulo compared to those from Minas Gerais. Furthermore, three features marked in blue were significantly more intense in samples from Minas Gerais, corresponding to features 23, 191, and 209.

Although the volcano plot indicated four significantly different features between the regions, 223 features were not significantly different and are shown in gray in Figure 1C. The features positioned closest to zero, circled in red, corresponded to the smallest differences observed between the samples (29, 72, 129, 185 and 221). Of these, only β-alaskene (feature 185) was identified. Figure S6 presents some of the chromatograms of propolis samples of this species from both regions, marking the features that showed significantly different intensities. Nt2J sample, from the São Paulo region, presented an intense peak related to feature 28 (Rt 4.67 min), while the other peaks remained close to the baseline. As the chromatograms were normalized by the peaks of greatest intensity, this apparent difference was accentuated, but statistical analyses showed that few differences were actually significant.

In general, these results indicate that, although there are some significant differences in the chemical composition of *Nannotrigona testaceicornis* propolis samples between the states of Minas Gerais and São Paulo, most of the features did not vary significantly. Of the 227 features evaluated, only four were considered significantly different, corresponding to approximately 1.76% of the total analyzed. As with the other three species, the chemical similarity between the samples outweighs the differences. Furthermore, this constitutes the first study related to the volatile composition of *Nannotrigona testaceicornis* propolis.

### 2.4. Comparison of Samples of Bee Species Within Each Region

To analyze the results from a complementary perspective, we compared samples from the same region grouped by bee species. In this way, we sought to verify the existence of differences in the composition of volatile compounds within the same region that may be associated with the bee species.

#### 2.4.1. Samples of (Geo)propolis from Jaguariúna (SP)

In Jaguariúna, propolis samples were collected from seven bee species: *Frieseomelitta varia* (Fv), *Melipona bicolor* (Mb), *Melipona marginata* (Mm), *Melipona quadrifasciata* (Mq), *Nannotrigona testaceicornis* (Nt), *Plebeia droryana* (Pd), and *Tetragonisca angustula* (Ta).

**Figure 2:**
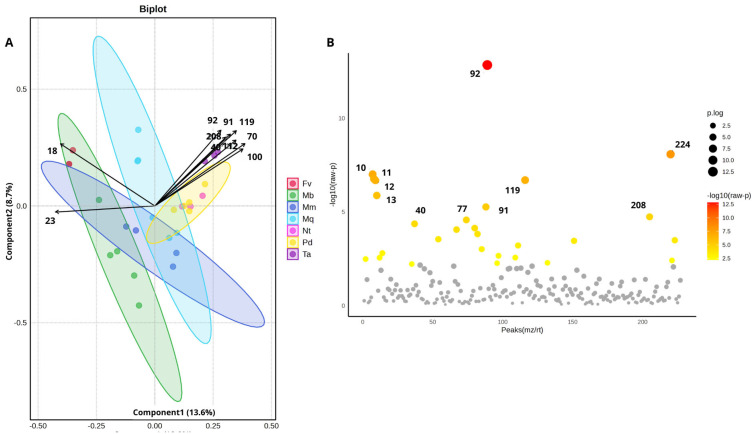
Multivariate analysis of the chemical profiles of bee species. (**A**) PLS-DA scores graph of the first two components, showing the distribution of samples and the features that contributed most to the discrimination between groups. (**B**) Analysis of variance (ANOVA) of samples collected in Jaguariúna—SP, highlighting the variables with significant differences between the species *Frieseomelitta varia* (Fv), *Melipona bicolor* (Mb), *Melipona marginata (*Mm*), Melipona quadrifasciata* (Mq), Nannotrigona testaceicornis (Nt), *Plebeia droryana* (Pd) and *Tetragonisca angustula* (Ta). The number of samples and names are described in Table 2. Accuracy: 0.39/R2:0.91/Q2:0.74.

Multivariate analysis using Partial Least Squares Discriminant Analysis (PLS-DA) (Figure 2A) revealed partial separation among the evaluated species, with the first two components explaining 22.3% of the total data variance (Component 1 = 13.6%; Component 2 = 8.7%). A tendency for the species *Plebeia droryana* (Pd), *Nannotrigona testaceicornis* (Nt), and *Tetragonisca angustula* (Ta) to cluster in the positive region of Component 1 was observed, while *Frieseomelitta varia* (Fv) and *Melipona bicolor* (Mb) showed a predominant distribution in the negative region of this axis. Features 92, 91, 119, 70, 100, 208, and 224 mainly contributed to the separation of the groups located in the positive region of Component 1, whereas features 18 and 23 were associated with the groups positioned in the negative region.

When we performed the analysis of variance (ANOVA) (Figure 2B), considering a significance level of *p* < 0.05, we observed that 57 features were significantly different between these propolis samples (shown in red, orange and yellow) and 170 features were not significantly different (shown in gray). In other words, of the 227 features, approximately 25% of the composition was significantly different between the species. ANOVA indicated that feature 92 (para-ethylphenol) was the most discriminating compound among the groups, followed by features 224, 119, 208, and 91, which were identified as trans-vettonal (224), silphinene (208), and estragole (91), suggesting that these metabolites play an important role in the chemical differentiation of samples from these species. Inversely, the features that showed the least differences were features 7, 50, 62, 74, 75, 79, 110, 150, 171, 193, 209, 210, and 229, with feature 210 being identified as trans-calamene. In this sense, although the local vegetation exerts an influence on the supply of resins for these bees, differences in the chemical composition of the resins are a result of a bee species’ choice of plants.

#### 2.4.2. Samples of (Geo)propolis from Cabo Verde and Muzambinho—MG

In Minas Gerais (Cabo Verde and Muzambinho), propolis samples were collected from five bee species: *Melipona quadrifasciata* (Mq), *Nannotrigona testaceicornis* (Nt), *Tetragonisca angustula* (Ta), *Melipona rufiventris* (Mr), and *Plebeia droryana* (Pd).

Partial Least Squares Discriminant Analysis (PLS-DA) separated groups of the evaluated species, mainly along Component 1. Species Mq showed a more distinct clustering pattern than the others, while considerable overlap was observed between some groups, indicating partial similarity in their chemical profiles. The first two components together explained 21.3% of the total variance of the data, suggesting that the observed discrimination represents only part of the existing variation among the species. Features 24, 13, 25, 48, 49, 60, 178, and 179 were mainly associated with the discrimination of *Melipona quadrifasciata* Mq from other groups, whereas feature 154 contributed to the grouping of the other species’ samples on the positive side of Component 1: (*Melipona rufiventris* (Mr), *Nannotrigona testaceicornis* (Nt), *Plebeia droryana* (Pd), and *Tetragonisca angustula* (Ta).

**Figure 3:**
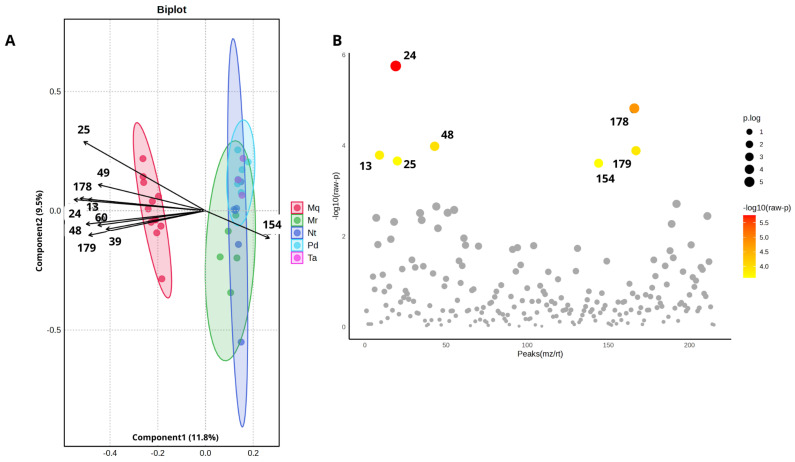
Multivariate analysis of the chemical profiles of bee species. (**A**) PLS-DA scores graph of the first two components, showing the distribution of samples and the features that contributed most to the discrimination between groups. (**B**) Analysis of variance (ANOVA) of samples collected in the State of Minas Gerais, Brazil (Cape Verde and Muzambinho), highlighting the variables with significant differences between the species *Melipona quadrifasciata* (Mq), *Nannotrigona testaceicornis* (Nt), *Tetragonisca angustula* (Ta), *Melipona rufiventris* (Mr), and *Plebeia droryana* (Pd). The number of samples and names are described in Table 2. Accuracy: 0.56/R2:0.72/Q2:0.63.

However, when we performed the analysis of variance (ANOVA) (Figure 3B), considering a significance level of *p* < 0.05, we observed that only seven features were significantly different between the propolis samples studied, and 220 features did not present significant differences. In other words, of the 227 features analyzed, approximately 3% of the composition showed a significant difference between species. The seven features that showed the greatest differences were: 13, 24, 25, 48, 154, 178 and 179. From these, the following compounds were identified: tricyclene (feature 24) and β-Selinene (feature 179).

These results show some significant differences in the composition of propolis samples among the different bee species studied in the Minas Gerais region (Muzambinho and Cabo Verde). It was also possible to observe through the ANOVA test (Figure 3B) that some features (24, 25, 48, 178, 179 and 204) were responsible for the clustering that occurred in the PLS-DA (Figure 3A) with the *Melipona quadrifasciata* (Mq) samples.

Once again, although the local vegetation exerts an influence on the supply of resins for these bees, differences in the chemical composition of the resins are a result of a bee species’ choice of plants.

## 3. Discussion

There are very few studies regarding volatile components of stingless bee propolis. In fact, none were found related to the volatile compounds of *Melipona quadrifasciata* propolis from the Southeast region of Brazil, but there are some studies of samples from the South of Brazil. Some studies analyzed the volatile composition of essential oil from *Melipona quadrifasciata* propolis obtained by hydrodistillation [7,9], while other authors [8] used the headspace technique for the analysis of volatile compounds. In addition, studies have been reported that evaluated the chemical profile of the volatile compounds of this propolis, although without specifying the extraction technique used [6]. Certainly, the variation in extraction methods affects the results. The extraction method developed by our group [10] and used herein is an attempt to standardize the extraction and analytical method in order to furnish reproducible results.

The low variability described by PCA limited conclusions about grouping and variability, indicating great similarity between samples. However, PCA analyses allowed a global visualization of the data and distribution of the samples. Thus, PCA was used exclusively as an exploratory tool (Supplementary Figures S1 and S2) and not as the sole basis for biological inferences. The interpretations of the chemical variability of samples in the present study were based on the integration of complementary approaches, including univariate analyses and volcano plots, as well as supervised multivariate analysis (PLS-DA). When comparing the volatile composition of samples from the Southeastern region (São Paulo and Minas Gerais) in this study with those reported for samples from the Southern region (Santa Catarina, Paraná, and Rio Grande do Sul) [6,7,8,9], some compounds were found in common, including tricyclene, α-pinene, α-terpinene, para-cymene, myrtenal, and alaskene. Despite these qualitative similarities, relevant variation was observed in their relative intensities.

In *Melipona quadrifasciata* samples from the states of Santa Catarina and Rio Grande do Sul, α-pinene and β-pinene were described as major compounds, presenting concentrations between 25.40% and 57.90% and between 5.89% and 28.08%, respectively [7,8,9]. In contrast, in the present study, involving 18 samples from the Southeast region, the features that presented concentrations greater than 3% and occurred in at least 50% of the samples corresponded to features 11, 12, 13, 24, 25, and 85, and only feature 24 was identified as tricyclene. The compound, α-pinene, was not observed as a major constituent in samples from the Southeast region, while β-pinene was detected only in low relative abundance, in contrast to previous studies in which it was reported as a major component. Similarly, Bankova et al. [6] also did not report high concentrations of α-pinene and β-pinene in samples from the state of Paraná. On the other hand, tricyclene, highlighted as the major compound in samples from the Southeast, was not reported as the major compound in samples from Southern Brazil. These differences in chemical composition may be related both to the extraction method used and to regional factors, especially the availability of local flora used by bees.

Some studies suggest species of the genus *Pinus* as a likely botanical source for *Melipona quadrifasciata* propolis, considering the wide occurrence of these plants in the Southern region of Brazil [7,9]. According to the authors, the state of Santa Catarina has approximately 29,000 hectares cultivated with *Pinus*, in addition to the fact that α-pinene can reach concentrations of up to 61.7% in the essential oils of these plant species. The authors also highlighted the predominance of *Pinus taeda* and *Pinus elliottii* in that region. According to the Brazilian Biodiversity Information System (SiBBr), these plant species have a wide distribution in the South and Southeast regions of Brazil, as does *Melipona quadrifasciata*.

Regarding *Tetragonisca angustula* propolis, a study analyzed the essential oil obtained by hydrodistillation of samples from the Southern region of Brazil and identified three monoterpenes. The major compound was α-pinene, with a relative concentration of 88.71%, followed by β-pinene (8.08%) and traces of tricyclene [7].

In the present study, however, a distinct chemical profile was observed for the samples from the Southeast region. Para-ethylphenol stood out as the main compound, reaching up to 50% in some samples. In addition, high concentrations of α-pinene and 2-epi-α-funebrene were identified, as well as features 32 and 141, which could not be identified. As discussed previously for *Melipona quadrifasciata*, these differences may be related to both the analytical methods employed and the influence of regional vegetation. The study suggests *Eucalyptus grandis* as a likely botanical source of *Tetragonisca angustula* propolis, due to the high concentrations of α-pinene present in the essential oils of this plant species [7].

To date, no studies have been found in the literature related to the volatile compounds present in *Plebeia droryana* propolis, nor in *Nannotrigona testaceicornis* propolis. This is the first study of the volatile composition of propolis from these species, making comparisons with previously published data unfeasible.

Analyzing the results of (geo)propolis samples from some bee species in the States of São Paulo and Minas Gerais and attempting to correlate them with studies available in the current literature, it was possible to identify some studies involving species of the Melipona genus. Analyzing the volatile composition of a geopropolis sample from the bee species, *Melipona seminigra*, originating from the state of Santa Catarina, the authors identified δ-cadinene (20.37%), β-oplopenone (12.80%), and α-copaene (11.94%) as the major compounds [9]. On the other hand, in the present work, the volatile composition of *Melipona seminigra* geopropolis from Minas Gerais showed higher intensities of features 27, 28, 30, 68, 80, 210 and 211, with the following features being identified: meta-cymenene (feature 80), trans-calamenene (feature 210) and cis-calamenene (feature 211).

The volatile compounds of geopropolis from *Melipona bicolor* were analyzed, with the following major compounds: α-pinene; β-pinene; limonene; terpinen-4-ol; α-copaene; β-caryophyllene and δ-cadinene [9]. Comparing this with the volatile composition of *M. bicolor* geopropolis samples from Jaguariúna (SP), we noted that the most intense features were 23, 24 and 25, with feature 25 being tricyclene, a very different composition, which may be related to the region.

In 1999, Bankova and colleagues identified ethylphenol (10.20%) as the compound present in the highest concentration in the essential oil of geopropolis from the bee species *Melipona compressipes* in Piauí. In addition, the authors identified the presence of several acids: isovaleric acid, caproic acid, mystic acid, palmitic acid and cinnamic acid. One of the geopropolis samples from *Melipona rufiventris* from Minas Gerais presented a high percentage of o-ethylphenol (33.63%) in its volatile composition, similar to the cited study [6].

As the extraction methods used in the literature and the region where the samples were collected are different from those reported herein, comparison with previous results is hindered. Another limitation of this study includes the relatively small and unbalanced number of replicates between species, as well as the restricted geographic coverage. It was not possible to standardize or predefine the number of samples per species and collection site, since the study depended on the availability of material provided by the partner meliponiculturists.

## 4. Materials and Methods

### 4.1. Samples

#### Sample Collection Site

The bee species were identified and samples provided by meliponiculturists from two Brazilian states (Minas Gerais: Cabo Verde and Muzambinho cities) and São Paulo (Jaguariúna city). Samples from 8 different species of native bees were studied, totaling 60 samples. The samples were collected by our group and immediately packaged in sealed containers and stored under refrigeration. Each sample was identified by an abbreviation that includes the species name, the sample number, and the region, as shown in Table 2 below. Although the samples were collected by the research group itself, it was not possible to standardize or predefine the number of samples per species and collection site. In spite of this limitation, this sample size is much larger than any other reported studies of Brazilian stingless propolis volatiles [5].

### 4.2. Sample Extraction Method

The samples were analyzed by headspace solid-phase microextraction (HS-SPME) using polydimethylsiloxane/divinylbenzene (PDMS/DVB) fiber (Supelco, Bellefonte, PA, USA), chosen for its ability to extract compounds of different polarities [10]. For extraction, 1 g of crushed sample was placed in a 20 mL headspace flask and incubated at 75 °C for 30 min. Then, the SPME fiber was exposed to the headspace for 20 min. After extraction, the fiber was inserted into the GC injector, with thermal desorption for 1 min at 230 °C in splitless mode.

### 4.3. Gas Chromatography Coupled to Mass Spectrometry, GC-MS

The analyses were conducted using a Thermo Scientific TRACE 1300 gas chromatograph coupled to a Thermo Scientific ISQ QD mass spectrometer (Thermo Scientific, Waltham, MA, USA). A fused silica capillary column HP-5ms (Agilent J&W, Santa Clara, CA, USA) with dimensions of 30 m × 0.25 mm and a film thickness of 0.25 μm was used. The oven temperature program started at 60 °C, increasing to 250 °C at a rate of 3 °C/min, with a final residence time of 3 min. Helium was used as the carrier gas, with a flow rate of 0.7 mL/min. Mass spectrometry detection was performed by electron ionization (70 eV) in positive mode, with full-scale scanning acquisition in the m/z 40–400 range.

### 4.4. Data Pre-Processing

The results obtained by GC-MS were processed using MS-DIAL 4 (2020) software, which performs peak extraction from the raw data, baseline filtering, followed by calibration, alignment, deconvolution, and peak integration [11]. Although MS-DIAL is a widely used and efficient tool for processing GC-MS data in untargeted metabolomics studies, its main limitation lies in its reliance on spectral libraries for compound identification, resulting in partial identification of detected features. On the other hand, the use of MS-DIAL was fundamental for processing a relatively large and diverse sample set (60 samples distributed across 8 different species), allowing for the efficient and standardized execution of critical steps such as peak detection, alignment, and deconvolution. This approach enabled the integrated analysis of a large number of samples with significant biological variability. This software also assists in substance identification. The amplitude used for peak detection was 306, with an average width of 20 min, and the database for identification associated with MS-Dial was the MassBank of North America (MoNA), considering only compounds with a similarity (score) greater than 70%. The confirmation of the compounds identified in the MS-DIAL was performed by calculating the linear retention indices, based on a series of standard alkanes, and by comparing the mass spectra with the Adams (2017) [12] library. Therefore, of the 227 detected features (*m*/*z* and retention time) only 76 were identified based on both MoNA and Adams (2017).

### 4.5. Chemometric Analysis of Data

Chemical composition data (both identified and unidentified features) were evaluated using different chemometric approaches. The data were initially normalized by sum and autoscaled, and then submitted to principal component analysis (PCA) and volcano plots for comparison between regions. Partial Least Squares Discriminant Analysis (PLS-DA) with cross-validation was performed, with parameters cited in the figures. Analysis of variance (ANOVA, *p* < 0.05) aimed to identify the most relevant features among the species and regions investigated. All analyses were performed on the MetaboAnalyst 6.0 platform [13]. No correction for multiple tests (e.g., false discovery rate (FDR)) was applied in this study. Considering some small and unbalanced groups, correction methods such as FDR can reduce statistical power, causing real differences to go undetected, potentially generating false negative results.

## 5. Conclusions

Analyses of (geo)propolis samples from São Paulo and Minas Gerais, collected from the same species of bee, indicated that, although some differences in volatile composition were observed, most compounds were similar. These results indicate that the same bee species collects similar resins in different regions, if they are available.

When comparing samples within the same region, separated by bee species, we sought to identify possible differences in volatile composition associated with the bee species. In the state of São Paulo (Jaguariúna), 25% of the compounds were significantly different between species.

In Minas Gerais (Muzambinho and Cabo Verde), the results also showed some significant differences in volatile composition; 12 features were significantly different between the samples, corresponding to approximately 5% of the composition. This was especially notable for the *Melipona quadrifasciata* (Mq) samples. However, for other samples, most of the features did not present significant differences. These results indicate that, although the local vegetation determines the supply of resins for these bees, differences in the chemical composition of propolis are a result of a species’ choice of plant species.

## Figures and Tables

**Table 1:** Compounds identified in GC-MS analyses numbered according to the list of features with retention time (RT), calculated retention index (RI), theoretical retention index (AI), molecular formula and average percentage in the samples. Numbers in bold indicate components higher than 1% in each chromatogram.

N°	RT (min)	Compound	RI.	AI	Formula	MqJ	MqCV	MqM	TaJ	TaM	NtJ	NtM	PdCV	PdJ	FvJ	MrCV	MbJ	MmJ
14 24 33	3.99	α-Thujene	924	924	C10H16	-	**2.17**	0.06	0.02	0.01	0.01	0.01	0.01	0.02	0.03	-	0.32	0.02
24	4.37	Tricyclene	911	921	C10H16	**1.07**	**4.71**	**4.05**	0.01	0.01	**2.59**	0.01	0.01	**1.56**	**6.92**	-	**6.21**	**2.34**
33	5.10	α-Pinene	936	932	C10H16	0.12	0.54	**1.06**	**2.56**	**4.22**	**3.15**	**2.17**	**1.22**	**4.09**	0.11	**2.73**	0.35	0.46
38 40	5.42	Camphene	946	946	C10H16	0.14	0.95	**1.13**	0.04	0.02	**1.60**	0.02	0.25	0.71	0.01	0.01	0.33	0.21
40	5.65	Sabinene	969	969	C10H6	0.01	**2.48**	**3.26**	0.01	0.06	0.01	0.06	0.01	0.09	-	0.03	-	-
45 49 51 52	5.99	tert-Butylbenzene	969	976	C10H14	0.05	-	-	0.01	0.01	0.02	0.01	-	-	0.05	-	0.12	0.41
49	6.47	β-Pinene	960	974	C10H16	0.29	0.45	0.70	0.01	0.01	0.01	0.01	0.01	0.01	0.01	0.03	0.44	0.50
51	6.58	β-Myrcene	986	988	C10H16	0.05	0.01	0.21	0.03	0.02	0.02	0.01	0.22	0.01	0.03	0.01	0.51	0.01
52	6.66	δ-2-Carene	994	1001	C10H16	-	0.20	0.03	0.01	0.01	0.03	0.01	0.01	0.01	0.12	0.01	0.03	0.05
58 60	6.96	δ-3-Carene	1006	1008	C10H16	0.01	0.10	0.01	0.01	0.01	0.01	0.08	0.02	0.05	-	0.01	0.03	0.01
60	7.18	α-Terpinene	1010	1014	C10H16	0.24	0.67	0.63	0.29	0.43	0.06	0.21	0.15	0.18	-	0.19	0.84	0.66
65	7.42	p-Cymene	1014	1020	C10H14	0.46	0.02	**2.55**	0.02	0.02	0.04	0.04	0.03	0.03	**2.60**	0.02	**1.91**	**2.01**
66	7.44	Limonene	1015	1024	C10H16	0.40	0.07	0.85	0.03	0.02	0.02	0.05	0.19	0.05	0.05	0.11	0.54	0.28
69	7.63	o-Cymene	1023	1022	C10H14	0.02	0.01	0.06	0.02	0.34	**4.32**	0.10	0.77	**1.73**	0.27	0.02	**1.80**	**5.83**
76	8.57	γ-Terpinene	1051	1054	C10H16	0.16	0.28	0.34	0.03	0.01	-	0.02	0.38	0.04	-	0.01	0.29	0.22
77	8.6	α-Methylbenzyl alcohol	1051	1057	C8H10O	**1.06**	0.19	0.09	0.03	0.02	0.01	0.01	0.02	0.01	0.39	0.01	0.08	0.06
78	9.39	Terpinolene	1076	1086	C10H16	-	-	0.17	0.01	0.01	0.01	0.03	-	0.01	-	0.01	0.19	-
80	9.85	m-Cymenene	1086	1082	C10H12	0.05	-	-	0.02	0.01	0.01	0.01	-	-	-	0.01	0.58	0.52
81	10.84	Benzaldehyde dimethyl acetal	1117	1109	C9H12O2	**1.70**	-	0.13	0.01	0.01	0.01	0.01	-	-	-	0.01	-	0.01
83	13.45	Ethyl benzoate	1180	1169	C9H10O2	0.57	0.30	0.07	0.03	0.02	0.01	0.03	0.04	0.01	0.04	0.01	-	-
84	13.51	Borneol	1183	1165	C10H18O	0.02	0.03	0.32	0.02	0.05	0.02	0.03	0.01	0.01	0.01	0.03	0.40	0.02
88	13.78	o-Ethylphenol	1190	1178	C8H10O	**5.68**	**4.33**	**4.61**	0.02	0.01	0.01	0.01	-	-	-	**6.74**	0.01	-
91	13.9	Estragole	1192	1195	C10H12O	0.01	-	0.04	0.01	0.01	-	-	-	-	-	-	-	-
92	13.96	p-Ethylphenol	1187	-	C8H10O	0.17	**1.48**	**1.40**	**37.66**	**5.41**	0.01	0.01	**4.57**	**3.16**	0.06	**7.7**	0.37	0.01
94	14.25	p-Methylacetophenone	1196	1190	C9H10O	-	0.02	0.10	0.02	0.04	0.02	0.05	0.01	0.01	0.04	0.01	0.23	0.02
97	14.42	p-Cymen-9-ol	1206	1204	C10H14O	0.03	0.02	0.23	0.03	0.06	0.01	0.01	0.21	0.01	0.47	0.01	**1.00**	0.28
98	14.53	Myrtenal	1209	1195	C10H14O	-	0.03	0.42	0.03	0.03	0.01	0.02	0.04	0.01	-	0.05	0.15	0.06
101	14.74	α-Terpineol	1214	1186	C10H18O	0.13	0.01	0.30	0.21	0.28	0.13	0.16	0.10	0.07	0.02	0.07	0.20	0.06
104	15.3	Isobornyl formate	1230	1235	C11H18O2	-	-	0.09	0.01	0.05	**1.83**	0.01	-	-	-	0.01	-	-
106	15.67	Thymol methyl ether	1237	1232	C11H16O	-	0.07	0.51	0.03	0.01	0.06	0.01	-	0.03	0.04	0.01	0.15	0.06
108	15.77	Carvacrol methyl ether	1241	1241	C11H16O	0.03	0.05	0.30	0.02	0.05	0.08	0.01	0.02	0.10	0.01	-	**2.41**	0.14
115	18.85	Silphiperfol-5-ene	1319	1326	C15H24	-	0.99	-	-	-	-	-	-	-	-	-	-	-
116	19.11	δ-Elemene	1326	1335	C15H24	0.01	0.03	0.05	0.02	0.02	0.01	0.02	0.27	0.15	**1.03**	0.19	0.07	0.17
119	19.45	Silphinene	1335	1345	C15H24	-	0.30	-	0.01	0.01	-	-	-	-	0.03	-	-	-
123	20.08	Carvacrol acetate	1355	1370	C12H16O2	-	0.02	0.02	0.02	0.01	0.01	0.04	0.10	-	0.01	0.01	0.01	-
124	20.15	α-Cubebene	1357	1348	C15H24	0.01	0.78	0.27	0.02	0.01	0.01	0.03	0.02	0.01	0.03	0.15	0.66	0.02
128	20.38	Benzyl α-methylbutyrate	1359	1363	C12H16O2	0.17	0.06	0.26	0.04	0.08	0.05	**1.40**	0.80	0.03	0.05	0.09	0.65	0.55
132	20.75	α-Ylangene	1370	1373	C15H24	0.58	0.82	0.10	0.19	0.01	0.01	**1.22**	0.03	0.04	0.35	0.02	0.13	0.07
135	21.04	β-Bourbonene	1376	1387	C15H24	0.09	0.31	0.01	0.03	0.03	0.03	0.30	0.15	0.05	0.08	0.03	0.18	0.18
136	21.12	α-Copaene	1380	1374	C15H24	0.63	0.37	0.26	0.02	0.02	0.02	**1.49**	**1.08**	0.01	0.36	0.02	0.93	**1.21**
137	21.16	β-Elemene	1381	1389	C15H24	0.63	0.34	0.24	0.02	0.02	0.01	0.09	**1.77**	-	0.53	0.01	**1.17**	**1.44**
140	21.44	2-epi-α-Funebrene	1386	1380	C15H24	0.19	0.34	0.20	**2.23**	**7.23**	**1.18**	**2.99**	**2.68**	**1.00**	0.30	**3.22**	0.13	0.31
145	21.76	Sibirene	1395	1400	C15H24	0.09	0.01	0.11	0.02	0.01	0.01	0.28	-	0.01	0.60	0.01	0.10	0.02
148	21.95	β-Longipinene	1401	1400	C15H24	0.20	0.09	0.03	0.04	0.12	0.01	0.12	0.38	0.01	-	0.17	0.04	0.08
149	22.14	α-Cedrene	1401	1410	C15H24	0.21	0.27	0.11	0.12	**1.00**	0.16	0.38	0.15	0.48	0.35	0.23	0.10	0.09
159	23.25	β-Copaene	1434	1430	C15H24	0.30	0.01	0.13	0.70	**1.84**	**1.79**	0.58	0.16	0.22	0.64	0.79	0.04	0.27
161	23.49	trans-Caryophyllene	1427	1417	C15H24	0.02	0.11	0.08	0.70	0.03	0.29	0.61	0.05	0.07	0.30	0.04	0.04	0.26
162	23.74	α-Himachalene	1446	1449	C15H24	0.09	0.04	0.15	0.20	**1.46**	0.38	0.95	0.84	**1.20**	0.43	0.97	0.23	0.12
164	23.87	α-trans-Bergamotene	1435	1432	C15H24	0.05	0.04	0.15	0.05	0.09	0.04	0.08	0.03	0.11	0.28	0.05	0.05	0.08
166	23.97	Alloaromadendrene	1453	1458	C15H24	0.25	0.12	0.07	0.04	0.03	0.01	0.03	0.04	-	0.45	0.15	0.25	0.09
167	23.98	α-Humulene	1453	1452	C15H24	0.37	0.17	0.30	0.07	0.06	0.04	0.10	0.17	0.01	**2.43**	0.23	0.18	0.01
168	24.10	β-trans-Farnesene	1457	1454	C15H24	0.24	0.02	0.06	0.08	0.10	0.02	0.15	0.12	0.08	0.04	0.56	0.04	0.02
172	24.64	Dauca-5,8-diene	1469	1471	C15H24	0.66	0.19	0.06	0.06	**1.50**	0.01	**1.03**	0.52	0.15	**1.49**	0.95	0.07	0.22
175	24.88	Amorpha-4,7(11)-diene	1472	1479	C15H24	0.55	0.24	0.42	0.59	0.27	0.08	0.39	0.11	0.25	0.99	0.12	0.48	0.45
179	25.09	β-Selinene	1487	1489	C15H24	0.27	0.47	0.25	0.03	0.03	0.01	0.29	0.04	0.01	0.14	0.06	0.58	0.35
182	25.22	α-Amorphene	1480	1483	C15H24	0.49	0.17	0.23	0.27	**1.83**	0.17	**1.25**	**1.44**	0.30	0.32	0.67	0.70	**1.02**
185	25.36	β-Alaskene	1492	1498	C15H24	0.77	0.10	0.03	0.18	0.36	0.08	0.26	0.11	0.08	0.72	0.11	0.02	0.09
187	25.43	Muurola-4(14),5-diene	1492	1493	C15H24	0.45	0.11	0.50	**1.34**	0.14	0.08	**2.63**	**2.2**	0.16	0.04	0.27	**1.59**	0.05
189	25.52	δ-Selinene	1494	1492	C15H24	0.31	0.32	0.20	0.04	**1.17**	0.01	**2.92**	**2.64**	0.05	**1.02**	0.05	0.37	0.51
197	25.88	β-Bisabolene	1505	1505	C15H24	0.34	0.02	0.03	0.16	0.16	0.04	0.12	0.06	0.08	0.41	0.20	0.04	0.05
198	25.94	α-Muurolene	1490	1500	C15H24	0.25	0.15	0.55	**1.43**	**1.00**	0.20	0.63	0.18	0.37	0.84	0.97	0.46	0.13
199	25.96	β-Curcumene	1500	1514	C15H24	0.13	0.11	0.20	0.79	0.52	0.10	0.37	0.22	0.18	0.51	0.39	0.25	0.22
203	26.28	γ-Cadinene	1510	1513	C15H24	0.76	0.1	0.57	0.62	0.15	0.13	0.23	0.03	0.09	**1.03**	0.27	0.41	**1.14**
205	26.47	α-Alaskene	1521	1512	C15H24	0.77	0.08	0.08	0.04	0.18	0.01	0.09	0.11	0.09	0.01	0.05	0.36	0.24
206	26.64	δ-Cadinene	1514	1522	C15H24	0.13	0.11	0.13	0.07	0.01	0.01	0.26	0.28	0.05	0.01	0.39	0.30	0.50
210	26.94	trans-Calamenene	1516	1521	C15H22	0.82	0.11	0.52	0.21	0.06	0.02	0.09	**2.94**	0.89	**1.91**	0.01	**1.34**	**1.13**
211	26.99	cis-Calamenene	1535	1528	C15H22	**1.12**	0.11	0.62	0.03	0.02	0.03	0.04	**2.85**	0.87	**1.91**	**1.27**	**1.32**	**3.06**
213	27.09	trans-Cadina-1,4-diene	1537	1533	C15H24	0.25	-	0.02	0.34	0.12	0.04	0.11	0.04	0.04	0.64	0.11	0.04	0.01
215	27.32	α-Cadinene	1540	1537	C15H24	0.25	0.03	0.09	0.17	0.02	0.01	0.02	0.07	-	0.65	0.12	0.12	0.33
216	27.54	Selina-3,7(11)-diene	1550	1545	C15H24	0.22	0.03	0.07	0.07	0.10	0.04	0.18	0.13	0.04	0.31	0.23	0.09	0.19
217	27.55	α-Calacorene	1554	1544	C15H20	0.04	0.01	0.02	0.02	0.06	0.01	0.02	0.32	0.06	0.51	0.15	0.07	0.32
218	27.71	α-Calamenene	1555	1544	C15H20	0.53	0.05	0.26	0.01	-	-	0.01	-	-	0.28	0.01	0.60	0.56
219	27.79	β-Calamenene	1557	1564	C15H20	0.61	0.18	0.31	0.32	0.30	0.07	0.15	0.23	0.32	-	**1.16**	0.70	0.82
224	30.05	trans-Vettonal	1557	1555	C14H22O	-	-	-	0.08	0.09	0.09	0.11	0.02	0.06	0.64	0.01	0.02	0.03
228	32.38	Cadalene	1686	1675	C15H18	0.18	0.01	0.10	-	-	0.01	-	-	-	0.12	-	0.28	0.35
231	36.11	Benzyl benzoate	1802	1759	C14H12O2	0.58	0.01	0.21	0.03	0.02	0.04	0.01	0.01	0.01	0.36	0.01	-	0.74

MqJ: *Melipona quadrifasciata*—Jaguariúna/MqCV: *Melipona quadrifasciata*—Cabo Verde/MqM: *Melipona quadrifasciata*—Muzambinho/TaJ: *Tetragonisca angustula*—Jaguariúna/TaM: *Tetragonisca angustula*—Muzambinho/MrCV: *Melipona rufiventris*—Cabo Verde/NtJ: *Nannotrigona testaceicornis*—Jaguariúna/NtM: *Nannotrigona testaceicornis*—Muzambinho/PdCV: *Plebeia droryana*—Cabo Verde/PdJ: *Plebeia droryana*—Jaguariúna/FvJ: *Frieseomelitta varia*—Jaguariúna/MbJ: *Melipona bicolor*—Jaguariúna/MmJ: *Melipona marginata*—Jaguariúna.

## Data Availability

The data is available from the corresponding author upon reasonable request.

## Supplementary Materials

The following supporting information can be downloaded at: https://www.mdpi.com/article/10.3390/molecules31132363/s1, Table S1: Compounds identified in GC-MS analyses numbered according to the list of features (supplementary) with retention time (RT), calculated retention index (RI), theoretical retention index (AI), molecular formula and average percentage in the samples; Figure S1: Principal Component Analysis (PCA) was performed using samples collected from the regions of Jaguariúna (J), Campo Verde (CV), Muzambinho (M), and quality control (QC) samples; Figure S2: Principal Component Analysis (PCA) based on the chromatographic profiles obtained by GC–MS from (geo)propolis samples collected in Southeastern Brazil (states of São Paulo, SP, and Minas Gerais, MG). (A) *Melipona quadrifasciata*; (B) *Tetragonisca angustula*; (C) *Plebeia droryana* and (D) *Nannotrigona testaceicornis*. The plots show the distribution of samples according to the first two principal components (PC1 and PC2); Figure S3: Chromatograms of propolis samples produced by *M. quadrifasciata* (Mq1M, sample from Muzambinho; Mq2CV, sample from Cabo Verde; and Mq1J, sample from Jaguariúna); Figure S4: Chromatograms of propolis samples produced by *T. angustula*, collected in the Brazilian states of São Paulo (Ta2J) and Minas Gerais (Ta2M and Ta3M); Figure S5: Chromatograms of propolis samples produced by *P. droryana* (Pd1CV and Pd5CV, collected in the Cabo Verde region, Minas Gerais, MG; and Pd4J, collected in the Jaguariúna region, São Paulo, SP, Brazil); Figure S6: Chromatograms of propolis samples produced by *N. testaceicornis* (Nt2J, collected in Jaguariúna, São Paulo, SP; and Nt5M and Nt6M, collected in Muzambinho, Minas Gerais, MG, Brazil).

## Abbreviations

The following abbreviations are used in this manuscript:

GC-MS	Gas Chromatography–Mass Spectrometry
GC	Gas Chromatography
HS-SPME	Headspace Solid-Phase Microextraction
SPME	Solid-Phase Microextraction
PDMS/DVB	Polydimethylsiloxane/Divinylbenzene
MoNA	MassBank of North America
MS-DIAL	Mass Spectrometry—Data Independent AnaLysis
NIST	National Institute of Standards and Technology
RI	Calculated Retention Index
AI	Theoretical Retention Index
RT	Retention Time
MqJ	*Melipona quadrifasciata—*Jaguariúna
MqCV	*Melipona quadrifasciata—*Cabo Verde
MqM	*Melipona quadrifasciata—*Muzambinho
TaJ	*Tetragonisca angustula*—Jaguariúna
TaM	*Tetragonisca angustula*—Muzambinho
MrCV	*Melipona rufiventris—*Cabo Verde
MsM	*Melipona seminigra—*Muzambinho
NtJ	*Nannotrigona testaceicornis—*Jaguariúna
NtM	*Nannotrigona testaceicornis—*Muzambinho
PdCV	*Plebeia droryana—*Cabo Verde
PdJ	*Plebeia droryana—*Jaguariúna
TcCV	*Tetragona clavipes—*Cabo Verde
FsM	*Friesella schrottkyi—*Muzambinho
FvJ	*Frieseomelitta varia*—Jaguariúna
MbJ	*Melipona bicolor*—Jaguariúna
MmJ	*Melipona marginata*—Jaguariúna
Fv	*Frieseomelitta varia*
Fs	*Friesella schrottkyi*
Mb	*Melipona bicolor*
Mm	*Melipona marginata*
Ms	*Melipona seminigra*
Mq	*Melipona quadrifasciata*
Mr	*Melipona rufiventris*
Nt	*Nannotrigona testaceicornis*
Pd	*Plebeia droryana*
Ta	*Tetragonisca angustula*
Tc	*Tetragona clavipes*
PCA	Principal Component Analysis
PLS-DA	Partial Least Squares Discriminant Analysis
PC1	Principal Component 1
PC2	Principal Component 2
ANOVA	Analysis of Variance
FDR	False Discovery Rate
QCs	Quality Control Samples
MG	Minas Gerais
SP	São Paulo
SiBBr	Brazilian Biodiversity Information System
EMBRAPA	Empresa Brasileira de Pesquisa Agropecuária
