# Supplementary material for: Volatile Composition of Brazilian Stingless Bee Propolis
Source: Molecules. 2026 Jul 5;31(13):2363. doi: 10.3390/molecules31132363 (PMC13363534; doi:10.3390/molecules31132363)
Supplement: Supplementary file 1 [file molecules-31-02363-s001.zip › molecules-4387503-supplementary.pdf]

Table S1. Compounds identified in GC-MS analyses numbered according to the list of *features* (supplementary) with retention time (RT), calculated retention index (RI), theoretical retention index (AI), molecular formula and average percentage in the samples.

N°	RT (min)	Compound	RI.	AI	Formula	MqJ	MqCV	MqM	MrCV	NtJ	NtM	PdCV	PdJ	FvJ	MbJ	MmJ	TaJ	TaM
4	2.91	*unidentified*	-	-	-	0.00	0.00	0.29	1.01	0.11	2.37	0.68	0.60	0.00	0.00	0.00	0.35	0.09
5	3.03	*unidentified*	-	-	-	0.06	0.00	0.40	0.75	0.04	1.47	0.54	0.36	0.07	0.05	0.00	0.73	0.13
6	3.78	*unidentified*	-	-	-	0.00	3.42	0.00	0.00	0.01	0.01	0.00	0.00	0.00	0.00	0.00	0.01	0.01
7	3.80	*unidentified*	-	-	-	1.11	3.49	0.00	0.00	0.00	0.01	0.00	0.00	0.00	0.00	0.00	0.01	0.01
8	3.82	*unidentified*	-	-	-	0.00	0.01	0.02	0.00	0.01	0.01	0.00	0.00	0.01	0.23	0.00	0.02	0.01
9	3.83	*unidentified*	-	-	-	2.02	2.20	0.50	0.01	0.04	0.01	0.00	0.01	0.01	0.01	0.00	0.02	0.01
10	3.87	*unidentified*	-	-	-	4.93	1.84	1.16	0.01	0.00	0.01	0.00	0.00	0.00	0.00	0.00	0.01	0.01
11	3.92	*unidentified*	-	-	-	4.88	0.05	2.64	0.01	0.01	0.01	0.00	0.00	0.04	0.00	0.00	0.01	0.01
12	3.92	*unidentified*	-	-	-	9.77	0.04	4.47	0.00	0.00	0.01	0.00	0.00	0.07	0.00	0.00	0.01	0.00
13	3.96	*unidentified*	-	-	-	7.75	2.38	5.22	0.00	1.14	0.00	0.00	0.00	0.00	0.05	0.00	0.01	0.01
14	3.99	α-Thujene	924	924	C10H16	0.00	2.17	0.06	0.00	0.01	0.01	0.01	0.02	0.03	0.32	0.02	0.02	0.01
15	4.03	*unidentified*	-	-	-	3.33	3.86	2.85	6.67	1.34	3.07	1.91	0.33	0.00	0.02	0.15	1.01	2.14
16	4.08	*unidentified*	-	-	-	0.57	1.26	0.79	1.52	0.06	0.71	0.44	0.10	1.23	0.01	0.04	0.19	0.44
17	4.08	*unidentified*	-	-	-	0.02	1.39	1.14	0.00	0.00	0.01	0.00	0.01	3.43	0.05	0.00	0.02	0.01
18	4.13	*unidentified*	-	-	-	0.01	0.01	0.21	0.00	0.00	0.00	0.00	0.00	0.79	0.24	0.05	0.01	0.00
19	4.16	*unidentified*	-	-	-	0.00	0.05	1.67	0.00	0.00	0.01	0.00	0.00	4.02	1.90	0.08	0.02	0.01
20	4.16	*unidentified*	-	-	-	0.00	0.02	1.10	0.01	0.01	0.01	0.01	0.01	2.36	1.52	0.03	0.03	0.01
21	4.26	*unidentified*	-	-	-	0.27	3.59	1.71	0.00	0.00	0.00	0.00	0.07	3.83	4.08	1.22	0.02	0.01
22	4.28	*unidentified*	-	-	-	0.05	0.26	0.15	0.00	0.00	0.00	0.00	0.04	0.30	0.42	0.15	0.01	0.00
23	4.31	*unidentified*	-	-	-	0.13	4.46	1.78	0.01	0.01	0.01	0.00	0.02	4.35	3.62	0.87	0.02	0.01
24	4.37	Tricyclene	911	921	C10H16	1.07	4.71	04.05	0.00	2.59	0.01	0.01	1.56	6.92	6.21	2.34	0.01	0.01
25	4.50	*unidentified*	-	-	-	1.41	4.64	5.33	0.01	9.54	0.01	0.01	6.75	1.21	7.58	2.32	0.01	0.01
26	4.65	*unidentified*	-	-	-	1.12	0.28	1.53	0.00	10.49	0.00	0.38	11.14	0.16	2.55	1.16	0.01	0.01
27	4.77	*unidentified*	-	-	-	0.57	0.89	0.68	0.00	7.84	0.07	4.64	13.22	0.39	0.48	5.00	0.01	0.03
28	4.83	*unidentified*	-	-	-	0.48	0.85	01.06	0.01	9.39	0.08	5.12	9.87	0.10	1.91	4.20	0.04	0.02
29	4.85	*unidentified*	-	-	-	0.01	0.15	0.22	0.01	0.87	0.48	0.90	1.51	0.04	0.27	0.59	0.02	0.01
30	4.89	*unidentified*	-	-	-	0.04	0.12	0.86	0.03	1.17	2.74	5.48	6.64	0.13	1.82	6.51	0.05	0.18

31	4.91	*unidentified*	-	-	-	0.02	0.11	0.56	0.04	6.29	1.92	3.46	2.51	0.09	01.08	4.15	0.05	0.17
32	5.00	*unidentified*	-	-	-	0.17	0.53	0.44	1.86	11.68	4.58	4.31	3.69	0.15	1.46	3.53	0.80	5.90
33	5.10	α-Pinene	936	932	C10H16	0.12	0.54	1.06	2.73	3.15	2.17	1.22	4.09	0.11	0.35	0.46	2.56	4.22
34	5.16	*unidentified*	-	-	-	0.05	0.49	1.13	1.31	0.11	1.15	0.42	1.82	0.88	0.59	0.04	1.82	1.12
35	5.17	*unidentified*	-	-	-	0.01	0.18	0.23	0.40	0.02	0.14	0.06	0.44	0.24	0.24	0.05	0.44	0.22
36	5.20	*unidentified*	-	-	-	0.03	0.90	0.58	0.24	0.05	0.07	0.01	0.57	0.40	0.35	0.11	0.20	0.01
37	5.30	*unidentified*	-	-	-	0.08	0.00	0.37	0.01	0.56	0.01	0.01	0.08	0.78	0.44	0.18	0.01	0.01
38	5.42	Camphene	946	946	C10H16	0.14	0.95	1.13	0.01	1.60	0.02	0.25	0.71	0.01	0.33	0.21	0.04	0.02
39	5.45	*unidentified*	-	-	-	0.03	1.14	01.05	0.02	0.94	0.02	0.13	0.22	0.00	0.18	0.18	0.03	0.03
40	5.65	Sabinene	969	969	C10H6	0.01	2.48	3.26	0.03	0.01	0.06	0.01	0.09	0.00	0.00	0.00	0.01	0.06
41	5.77	*unidentified*	-	-	-	0.00	2.23	2.13	0.01	0.02	0.01	0.00	0.01	1.10	0.00	0.00	0.02	0.01
42	5.95	*unidentified*	-	-	-	0.13	0.11	0.14	0.02	0.01	0.01	0.00	0.00	0.17	0.02	0.02	0.03	0.01
43	5.96	*unidentified*	-	-	-	0.17	0.04	0.17	0.02	0.02	0.03	0.01	0.01	0.47	0.23	0.03	0.04	0.02
44	5.97	*unidentified*	-	-	-	0.04	0.03	0.06	0.01	0.01	0.03	0.02	0.00	0.20	0.09	0.00	0.01	0.00
45	5.99	tert-Butylbenzene	969	976	C10H14	0.05	0.00	0.00	0.00	0.02	0.01	0.00	0.00	0.05	0.12	0.41	0.01	0.01
46	6.12	*unidentified*	-	-	-	0.19	0.08	0.31	0.01	1.02	0.66	1.00	0.54	0.02	1.02	1.02	0.01	0.05
47	6.21	*unidentified*	-	-	-	0.27	0.85	0.46	0.43	1.60	0.40	1.96	1.79	0.35	0.50	1.37	0.29	1.87
48	6.39	*unidentified*	-	-	-	0.22	0.51	1.06	0.46	0.01	0.12	0.01	0.36	1.59	0.03	0.06	0.57	0.26
49	6.47	β-Pinene	960	974	C10H16	0.29	0.45	0.70	0.03	0.01	0.01	0.01	0.01	0.01	0.44	0.50	0.01	0.01
50	6.47	*unidentified*	-	-	-	0.14	0.22	0.29	0.01	0.14	0.10	0.06	0.43	0.00	0.13	0.23	0.17	0.38
51	6.58	β-Myrcene	986	988	C10H16	0.05	0.01	0.21	0.01	0.02	0.01	0.22	0.01	0.03	0.51	0.01	0.03	0.02
52	6.66	δ-2-Carene	994	1.001	C10H16	0.00	0.20	0.03	0.01	0.03	0.01	0.01	0.01	0.12	0.03	0.05	0.01	0.01
53	6.69	*unidentified*	-	-	-	0.01	0.17	0.23	0.03	0.03	0.11	0.08	0.07	1.09	0.06	0.02	0.09	0.06
54	6.71	*unidentified*	-	-	-	0.07	0.82	0.58	0.08	0.16	0.47	0.45	0.58	3.19	0.26	0.08	0.40	0.43
55	6.80	*unidentified*	-	-	-	0.24	0.38	0.43	0.01	0.01	0.06	0.02	0.01	1.21	0.46	0.53	0.02	0.01
56	6.91	*unidentified*	-	-	-	0.18	0.60	0.49	0.01	0.01	0.04	0.03	0.56	0.04	0.99	0.40	0.03	0.03
57	6.95	*unidentified*	-	-	-	0.02	1.36	0.11	0.00	0.01	0.09	0.02	0.08	0.00	0.09	0.06	0.01	0.01
58	6.96	δ-3-Carene	1.006	1.008	C10H16	0.01	0.10	0.01	0.01	0.01	0.08	0.02	0.05	0.00	0.03	0.01	0.01	0.01
59	7.00	*unidentified*	-	-	-	0.08	0.98	0.24	0.00	0.00	0.65	0.52	0.55	0.01	0.84	0.04	0.01	0.00
60	7.18	α-Terpinene	1.010	1.014	C10H16	0.24	0.67	0.63	0.19	0.06	0.21	0.15	0.18	0.00	0.84	0.66	0.29	0.43
61	7.18	*unidentified*	-	-	-	0.02	2.20	0.42	0.08	0.04	0.06	0.02	0.04	0.01	1.52	0.06	0.10	0.03

62	7.28	*unidentified*	-	-	-	0.07	0.41	0.32	0.02	0.01	0.02	0.01	0.01	0.26	0.09	0.01	0.03	0.02
63	7.34	*unidentified*	-	-	-	0.63	0.22	1.95	0.01	0.01	0.01	0.00	0.00	0.31	3.00	1.79	0.01	0.01
64	7.36	*unidentified*	-	-	-	0.16	0.35	0.23	0.00	0.07	0.23	0.16	0.09	0.00	0.23	0.71	0.01	0.03
65	7.42	p-Cymene	1.014	1.020	C10H14	0.46	0.02	2.55	0.02	0.04	0.04	0.03	0.03	2.60	1.91	2.01	0.02	0.02
66	7.44	Limonene	1.015	1.024	C10H16	0.40	0.07	0.85	0.11	0.02	0.05	0.19	0.05	0.05	0.54	0.28	0.03	0.02
67	7.53	*unidentified*	-	-	-	0.34	0.04	0.77	0.01	0.04	0.41	0.10	0.21	0.66	0.53	0.28	0.18	0.27
68	7.54	*unidentified*	-	-	-	0.29	0.03	0.54	0.01	0.08	0.01	0.06	0.11	1.37	0.42	6.13	0.03	0.02
69	7.63	o-Cymene	1.023	1.022	C10H14	0.02	0.01	0.06	0.02	4.32	0.10	0.77	1.73	0.27	1.80	5.83	0.02	0.34
70	8.02	*unidentified*	-	-	-	0.00	0.03	0.13	0.01	0.01	0.03	0.01	0.01	0.04	0.04	0.02	0.02	0.02
71	8.17	*unidentified*	-	-	-	0.00	0.01	0.01	0.01	0.13	0.01	0.00	0.03	0.02	0.02	0.03	0.01	0.01
72	8.29	*unidentified*	-	-	-	0.02	0.20	0.20	0.00	0.02	0.01	0.00	0.22	0.00	0.15	0.00	0.02	0.01
73	8.29	*unidentified*	-	-	-	0.00	0.01	0.01	0.01	0.01	0.01	0.00	0.01	0.00	0.01	0.01	0.01	0.01
74	8.48	*unidentified*	-	-	-	0.07	0.20	0.10	0.02	0.02	0.18	0.07	0.05	0.00	0.15	0.01	0.10	0.07
75	8.51	*unidentified*	-	-	-	0.09	0.29	0.21	0.01	0.01	0.04	0.32	0.04	0.01	0.22	0.02	0.14	0.02
76	8.57	γ-Terpinene	1.051	1.054	C10H16	0.16	0.28	0.34	0.01	0.00	0.02	0.38	0.04	0.00	0.29	0.22	0.03	0.01
77	8.60	α-Methylbenzyl alcohol	1.051	1.057	C8H10O	1.06	0.19	0.09	0.01	0.01	0.01	0.02	0.01	0.39	0.08	0.06	0.03	0.02
78	9.39	Terpinolene	1.076	1.086	C10H16	0.00	0.00	0.17	0.01	0.01	0.03	0.00	0.01	0.00	0.19	0.00	0.01	0.01
79	9.46	*unidentified*	-	-	-	0.07	0.00	0.25	0.03	0.01	2.23	0.01	0.02	0.03	0.01	0.00	0.02	0.02
80	9.85	m-Cymenene	1.086	1.082	C10H12	0.05	0.00	0.00	0.01	0.01	0.01	0.00	0.00	0.00	0.58	0.52	0.02	0.01
81	10.84	Benzaldehyde dimethyl acetal	1.117	1.109	C9H12O2	1.70	0.00	0.13	0.01	0.01	0.01	0.00	0.00	0.00	0.00	0.01	0.01	0.01
82	12.41	*unidentified*	-	-	-	0.00	0.00	0.18	0.01	1.11	0.01	0.01	0.01	0.00	0.01	0.01	0.03	0.02
83	13.45	Ethyl benzoate	1.180	1.169	C9H10O2	0.57	0.30	0.07	0.01	0.01	0.03	0.04	0.01	0.04	0.00	0.00	0.03	0.02
84	13.51	Borneol	1.183	1.165	C10H18O	0.02	0.03	0.32	0.03	0.02	0.03	0.01	0.01	0.01	0.40	0.02	0.02	0.05
85	13.68	*unidentified*	-	-	-	12.96	2.12	1.18	0.06	0.02	0.01	0.00	0.01	0.00	0.02	0.02	0.01	0.02
86	13.70	*unidentified*	-	-	-	0.02	0.06	0.48	0.01	0.06	0.03	0.03	0.04	0.01	0.43	0.08	0.06	0.05
87	13.75	*unidentified*	-	-	-	0.00	0.17	0.11	0.01	0.02	0.07	0.02	0.01	0.00	0.48	0.00	0.04	0.05
88	13.78	o-Ethylphenol	1.190	1.178	C8H10O	5.68	4.33	4.61	6.74	0.01	0.01	0.00	0.00	0.00	0.01	0.00	0.02	0.01
89	13.78	*unidentified*	-	-	-	0.15	0.05	0.58	0.17	0.05	0.05	0.14	0.03	0.00	0.40	0.05	0.07	0.03
90	13.85	*unidentified*	-	-	-	0.11	0.07	0.42	1.50	0.04	0.03	0.07	0.01	0.00	0.09	0.38	0.03	0.14
91	13.90	Estragole	1.192	1.195	C10H12O	0.01	0.00	0.04	0.00	0.00	0.00	0.00	0.00	0.00	0.00	0.00	0.01	0.01
92	13.96	p-Ethylphenol	1.187	-	C8H10O	0.17	1.48	1.40	7.70	0.01	0.01	4.57	3.16	0.06	0.37	0.01	37.66	5.41

93	14.02	*unidentified*	-	-	-	0.01	0.02	0.15	1.23	1.59	0.03	0.53	01.02	0.33	0.25	0.72	0.10	0.03
94	14.25	p-Methylacetophenone	1.196	1.190	C9H10O	0.00	0.02	0.10	0.01	0.02	0.05	0.01	0.01	0.04	0.23	0.02	0.02	0.04
95	14.35	*unidentified*	-	-	-	0.01	0.01	0.17	1.26	0.32	1.90	0.27	0.28	0.38	0.44	0.28	0.39	0.87
96	14.35	*unidentified*	-	-	-	0.00	0.01	0.16	0.01	0.02	0.01	0.02	0.01	0.47	0.75	0.15	0.01	0.01
97	14.42	p-Cymen-9-ol	1.206	1.204	C10H14O	0.03	0.02	0.23	0.01	0.01	0.01	0.21	0.01	0.47	1.00	0.28	0.03	0.06
98	14.53	Myrtenal	1.209	1.195	C10H14O	0.00	0.03	0.42	0.05	0.01	0.02	0.04	0.01	0.00	0.15	0.06	0.03	0.03
99	14.53	*unidentified*	-	-	-	0.06	0.02	0.42	0.04	0.04	0.06	0.34	0.41	0.28	1.28	0.42	0.14	0.52
100	14.68	*unidentified*	-	-	-	0.00	0.02	0.08	0.02	0.06	0.03	0.01	0.01	0.03	0.04	0.01	0.02	0.04
101	14.74	α-Terpineol	1.214	1.186	C10H18O	0.13	0.01	0.30	0.07	0.13	0.16	0.10	0.07	0.02	0.20	0.06	0.21	0.28
102	14.75	*unidentified*	-	-	-	0.12	0.02	0.37	0.01	0.07	0.03	0.03	0.02	0.05	0.38	0.05	0.02	0.03
103	15.08	*unidentified*	-	-	-	0.00	0.00	0.00	0.01	0.01	0.01	0.01	0.01	0.00	0.00	0.01	0.01	0.01
104	15.30	Isobornyl formate	1.230	1.235	C11H18O2	0.00	0.00	0.09	0.01	1.83	0.01	0.00	0.00	0.00	0.00	0.00	0.01	0.05
105	15.37	*unidentified*	-	-	-	0.00	0.01	0.12	0.01	1.21	0.02	0.02	0.05	0.01	0.00	0.00	0.03	0.02
106	15.67	Thymol methyl ether	1.237	1.232	C11H16O	0.00	0.07	0.51	0.01	0.06	0.01	0.00	0.03	0.04	0.15	0.06	0.03	0.01
107	15.76	*unidentified*	-	-	-	0.01	0.01	0.08	0.00	0.00	0.00	0.00	0.03	0.08	0.84	0.07	0.01	0.00
108	15.77	Carvacrol methyl ether	1.241	1.241	C11H16O	0.03	0.05	0.30	0.00	0.08	0.01	0.02	0.10	0.01	2.41	0.14	0.02	0.05
109	15.89	*unidentified*	-	-	-	0.00	0.08	0.23	0.12	0.05	0.01	0.02	0.00	0.16	01.04	0.90	0.02	0.07
110	15.94	*unidentified*	-	-	-	0.29	0.10	0.44	0.00	0.05	0.00	0.00	0.47	0.14	0.75	0.80	0.01	0.01
111	17.57	*unidentified*	-	-	-	0.00	0.55	0.13	0.31	0.01	0.01	0.00	0.00	0.00	0.01	0.00	0.01	0.01
112	17.58	*unidentified*	-	-	-	0.00	0.22	0.05	0.12	0.00	0.00	0.01	0.00	0.00	0.01	0.00	0.01	0.01
113	17.64	*unidentified*	-	-	-	0.00	0.61	0.26	5.19	0.01	0.01	0.01	0.00	0.00	0.02	0.00	0.01	0.01
114	17.69	*unidentified*	-	-	-	0.12	0.27	0.34	8.51	0.08	02.04	1.15	0.09	0.18	0.02	0.01	10.16	0.01
115	18.85	Silphiperfol-5-ene	1.319	1.326	C15H24	0.00	0.99	0.00	0.00	0.00	0.00	0.00	0.00	0.00	0.00	0.00	0.00	0.00
116	19.11	δ-Elemene	1.326	1.335	C15H24	0.01	0.03	0.05	0.19	0.01	0.02	0.27	0.15	1.03	0.07	0.17	0.02	0.02
117	19.13	*unidentified*	-	-	-	0.06	0.12	0.02	0.26	0.03	0.04	0.14	0.06	0.50	0.02	0.08	0.03	0.04
118	19.13	*unidentified*	-	-	-	0.02	0.04	0.02	0.07	0.01	0.01	0.19	0.10	1.38	0.04	0.11	0.02	0.02
119	19.45	Silphinene	1.335	1.345	C15H24	0.00	0.30	0.00	0.00	0.00	0.00	0.00	0.00	0.03	0.00	0.00	0.01	0.01
120	19.55	*unidentified*	-	-	-	0.00	0.02	0.00	0.01	0.01	0.01	0.01	0.01	0.43	0.00	0.00	0.02	0.02
121	19.56	*unidentified*	-	-	-	0.00	0.11	0.01	0.01	0.01	0.03	0.03	0.01	0.90	0.00	0.00	0.02	0.02
122	19.75	*unidentified*	-	-	-	0.48	0.18	0.24	1.52	0.01	0.01	0.00	0.00	0.04	0.01	0.00	0.01	0.01
123	20.08	Carvacrol acetate	1.355	1.370	C12H16O2	0.00	0.02	0.02	0.01	0.01	0.04	0.10	0.00	0.01	0.01	0.00	0.02	0.01

124	20.15	α-Cubebene	1.357	1.348	C15H24	0.01	0.78	0.27	0.15	0.01	0.03	0.02	0.01	0.03	0.66	0.02	0.02	0.01
125	20.25	*unidentified*	-	-	-	0.02	0.15	0.05	0.08	0.03	0.76	0.02	0.01	0.00	0.11	0.24	0.03	0.07
126	20.31	*unidentified*	-	-	-	0.16	0.24	0.28	0.25	0.03	1.54	0.69	0.01	0.14	0.46	0.31	0.03	0.02
127	20.33	*unidentified*	-	-	-	0.15	0.00	0.22	0.13	0.06	1.56	0.34	0.03	0.01	0.56	0.37	0.04	0.11
128	20.38	Benzyl α-methylbutyrate	1.359	1.363	C12H16O2	0.17	0.06	0.26	0.09	0.05	1.40	0.80	0.03	0.05	0.65	0.55	0.04	0.08
129	20.49	*unidentified*	-	-	-	0.11	0.03	0.13	0.74	0.50	0.58	1.62	0.39	0.03	0.56	1.32	0.87	2.27
130	20.56	*unidentified*	-	-	-	0.01	0.31	0.10	0.03	0.03	0.03	0.03	0.02	0.34	0.06	0.01	0.04	0.08
131	20.63	*unidentified*	-	-	-	0.08	0.31	0.21	0.09	0.07	0.11	0.06	0.05	0.17	0.48	0.74	0.15	0.14
132	20.75	α-Ylangene	1.370	1.373	C15H24	0.58	0.82	0.10	0.02	0.01	1.22	0.03	0.04	0.35	0.13	0.07	0.19	0.01
133	20.85	*unidentified*	-	-	-	0.67	0.04	0.20	0.01	0.01	0.92	0.05	0.05	1.11	0.12	0.05	0.02	0.03
134	21.03	*unidentified*	-	-	-	0.10	0.69	0.08	0.03	0.04	0.39	0.11	0.03	0.13	0.15	0.18	0.05	0.04
135	21.04	β-Bourbonene	1.376	1.387	C15H24	0.09	0.31	0.01	0.03	0.03	0.30	0.15	0.05	0.08	0.18	0.18	0.03	0.03
136	21.12	α-Copaene	1.380	1.374	C15H24	0.63	0.37	0.26	0.02	0.02	1.49	1.08	0.01	0.36	0.93	1.21	0.02	0.02
137	21.16	β-Elemene	1.381	1.389	C15H24	0.63	0.34	0.24	0.01	0.01	0.09	1.77	0.00	0.53	1.17	1.44	0.02	0.02
138	21.17	*unidentified*	-	-	-	0.09	0.42	0.03	0.00	0.00	0.01	0.24	0.00	0.05	0.11	0.19	0.01	0.01
139	21.38	*unidentified*	-	-	-	0.36	0.11	0.36	0.46	0.14	0.24	0.61	0.11	0.40	0.21	0.03	0.06	0.73
140	21.44	2-epi-α-Funebrene	1.386	1.380	C15H24	0.19	0.34	0.20	3.22	1.18	2.99	2.68	1.00	0.30	0.13	0.31	2.23	7.23
141	21.52	*unidentified*	-	-	-	0.36	0.29	0.14	3.25	1.88	2.99	1.36	0.82	0.69	0.03	0.58	4.25	7.59
142	21.53	*unidentified*	-	-	-	0.28	0.27	0.10	1.58	0.67	0.94	0.52	0.35	0.50	0.01	0.24	1.81	2.74
143	21.58	*unidentified*	-	-	-	0.05	0.47	0.03	0.20	0.16	0.37	0.02	0.10	0.14	0.03	0.06	0.61	0.41
144	21.66	*unidentified*	-	-	-	0.02	0.58	0.25	0.47	0.32	2.02	0.03	0.44	0.17	0.18	0.13	2.51	0.46
145	21.76	Sibirene	1.395	1.400	C15H24	0.09	0.01	0.11	0.01	0.01	0.28	0.00	0.01	0.60	0.10	0.02	0.02	0.01
146	21.85	*unidentified*	-	-	-	0.45	0.21	0.17	0.03	0.02	0.59	0.04	0.01	0.16	0.05	0.05	0.04	0.01
147	21.88	*unidentified*	-	-	-	0.35	0.18	0.25	0.06	0.06	1.33	0.38	0.01	0.09	0.07	0.16	0.07	0.02
148	21.95	β-Longipinene	1.401	1.400	C15H24	0.20	0.09	0.03	0.17	0.01	0.12	0.38	0.01	0.00	0.04	0.08	0.04	0.12
149	22.14	α-Cedrene	1.401	1.410	C15H24	0.21	0.27	0.11	0.23	0.16	0.38	0.15	0.48	0.35	0.10	0.09	0.12	1.00
150	22.26	*unidentified*	-	-	-	0.12	0.17	0.10	0.02	0.02	0.08	0.02	0.01	0.13	0.07	0.02	0.04	0.08
151	22.27	*unidentified*	-	-	-	0.30	0.26	0.13	0.22	0.08	0.60	0.21	0.03	0.18	0.24	0.06	0.10	0.91
152	22.27	*unidentified*	-	-	-	0.22	0.24	0.11	0.17	0.07	0.43	0.15	0.02	0.15	0.17	0.04	0.11	0.59
153	22.28	*unidentified*	-	-	-	0.21	0.36	0.17	0.17	0.09	0.61	0.12	0.05	0.18	0.27	0.04	0.26	0.89
154	22.43	*unidentified*	-	-	-	0.00	0.02	0.01	0.01	0.02	0.03	0.02	0.02	0.02	0.01	0.01	0.03	0.03

155	22.47	*unidentified*	-	-	-	0.05	0.58	0.11	0.11	0.10	0.34	0.61	0.03	0.31	0.14	0.03	0.19	0.01
156	22.51	*unidentified*	-	-	-	0.09	0.06	0.17	0.05	0.01	0.89	1.99	0.01	0.28	0.39	0.35	0.44	0.01
157	23.00	*unidentified*	-	-	-	0.09	0.01	0.03	0.22	0.10	0.44	0.99	0.14	0.01	0.26	0.42	0.05	0.11
158	23.25	*unidentified*	-	-	-	0.22	0.00	0.01	0.03	0.01	0.01	0.01	0.01	0.05	0.01	0.01	0.02	0.02
159	23.25	β-Copaene	1.434	1.430	C15H24	0.30	0.01	0.13	0.79	1.79	0.58	0.16	0.22	0.64	0.04	0.27	0.70	1.84
160	23.36	*unidentified*	-	-	-	0.04	0.49	0.11	0.38	0.06	0.42	0.07	0.29	0.29	0.07	0.30	1.54	0.71
161	23.49	trans-Caryophyllene	1.427	1.417	C15H24	0.02	0.11	0.08	0.04	0.29	0.61	0.05	0.07	0.30	0.04	0.26	0.70	0.03
162	23.74	α-Himachalene	1.446	1.449	C15H24	0.09	0.04	0.15	0.97	0.38	0.95	0.84	1.20	0.43	0.23	0.12	0.20	1.46
163	23.79	*unidentified*	-	-	-	0.03	0.05	0.20	0.21	0.11	0.29	0.10	0.17	0.95	0.15	0.05	0.04	0.46
164	23.87	α-trans-Bergamotene	1.435	1.432	C15H24	0.05	0.04	0.15	0.05	0.04	0.08	0.03	0.11	0.28	0.05	0.08	0.05	0.09
165	23.87	*unidentified*	-	-	-	0.11	0.17	0.55	0.07	0.05	0.31	0.07	0.14	1.83	0.07	0.16	0.36	0.17
166	23.97	Alloaromadendrene	1.453	1.458	C15H24	0.25	0.12	0.07	0.15	0.01	0.03	0.04	0.00	0.45	0.25	0.09	0.04	0.03
167	23.98	α-Humulene	1.453	1.452	C15H24	0.37	0.17	0.30	0.23	0.04	0.10	0.17	0.01	2.43	0.18	0.01	0.07	0.06
168	24.10	β-trans-Farnesene	1.457	1.454	C15H24	0.24	0.02	0.06	0.56	0.02	0.15	0.12	0.08	0.04	0.04	0.02	0.08	0.10
169	24.24	*unidentified*	-	-	-	1.04	0.58	0.21	0.06	0.03	0.04	0.05	0.04	0.03	0.64	0.10	0.05	0.01
170	24.37	*unidentified*	-	-	-	0.13	1.10	0.29	1.11	0.03	0.69	1.51	0.21	0.91	1.27	0.21	0.02	0.05
171	24.49	*unidentified*	-	-	-	0.27	0.37	0.83	0.97	0.04	0.74	4.19	0.33	0.50	0.31	1.49	0.09	0.46
172	24.64	Dauca-5,8-diene	1.469	1.471	C15H24	0.66	0.19	0.06	0.95	0.01	01.03	0.52	0.15	1.49	0.07	0.22	0.06	1.50
173	24.67	*unidentified*	-	-	-	0.17	0.18	0.00	0.48	0.01	0.41	0.18	0.03	0.03	0.04	0.01	0.01	0.57
174	24.77	*unidentified*	-	-	-	0.74	0.33	0.04	0.38	0.11	0.89	0.23	0.29	1.28	0.23	0.02	0.32	1.74
175	24.88	Amorpha-4,7(11)-diene	1.472	1.479	C15H24	0.55	0.24	0.42	0.12	0.08	0.39	0.11	0.25	0.99	0.48	0.45	0.59	0.27
176	24.92	*unidentified*	-	-	-	0.24	0.11	0.12	0.44	0.34	0.98	0.04	0.82	0.23	0.12	0.22	3.19	0.19
177	24.99	*unidentified*	-	-	-	0.29	0.01	0.01	0.03	0.01	0.02	0.01	0.01	0.19	0.02	0.02	0.02	0.02
178	25.01	*unidentified*	-	-	-	0.43	0.62	0.41	0.19	0.04	0.09	0.01	0.01	0.19	0.70	0.26	0.08	0.04
179	25.09	β-Selinene	1.487	1.489	C15H24	0.27	0.47	0.25	0.06	0.01	0.29	0.04	0.01	0.14	0.58	0.35	0.03	0.03
180	25.22	*unidentified*	-	-	-	0.38	0.14	0.09	0.37	0.01	0.87	0.77	0.06	0.44	0.46	0.60	0.08	0.61
181	25.22	*unidentified*	-	-	-	0.32	0.16	0.04	0.14	0.02	0.59	0.48	0.06	0.49	0.10	0.21	0.17	0.63
182	25.22	α-Amorphene	1.480	1.483	C15H24	0.49	0.17	0.23	0.67	0.17	1.25	1.44	0.30	0.32	0.70	01.02	0.27	1.83
183	25.27	*unidentified*	-	-	-	0.06	0.01	0.07	0.04	0.02	0.05	0.03	0.01	0.01	0.09	0.42	0.03	0.08
184	25.30	*unidentified*	-	-	-	0.10	0.82	0.45	1.55	0.69	1.75	0.94	0.65	0.79	1.18	1.00	0.88	3.12
185	25.36	β-Alaskene	1.492	1.498	C15H24	0.77	0.10	0.03	0.11	0.08	0.26	0.11	0.08	0.72	0.02	0.09	0.18	0.36

186	25.39	*unidentified*	-	-	-	0.76	0.10	0.03	0.13	0.05	0.28	0.16	0.14	0.85	0.03	0.02	0.21	0.18
187	25.43	Muurola-4(14),5-diene	1.492	1.493	C15H24	0.45	0.11	0.50	0.27	0.08	2.63	2.20	0.16	0.04	1.59	0.05	1.34	0.14
188	25.44	*unidentified*	-	-	-	0.38	0.16	0.37	0.46	0.06	2.49	1.57	0.39	0.24	01.01	0.18	0.91	0.06
189	25.52	δ-Selinene	1.494	1.492	C15H24	0.31	0.32	0.20	0.05	0.01	2.92	2.64	0.05	01.02	0.37	0.51	0.04	1.17
190	25.53	*unidentified*	-	-	-	0.37	0.11	0.10	0.07	0.01	0.14	0.13	0.07	0.13	0.15	0.29	0.01	3.57
191	25.58	*unidentified*	-	-	-	0.33	0.13	0.11	0.90	0.34	2.07	1.47	1.10	0.53	0.18	0.47	0.03	1.51
192	25.59	*unidentified*	-	-	-	0.10	0.20	0.07	0.92	0.34	0.50	0.25	0.04	0.93	0.11	0.12	0.03	1.53
193	25.65	*unidentified*	-	-	-	0.21	0.12	0.09	2.92	0.36	2.83	0.90	0.65	0.52	0.03	0.42	0.05	4.25
194	25.69	*unidentified*	-	-	-	0.29	0.03	0.01	0.13	0.05	0.12	0.15	0.07	0.41	0.01	0.03	0.18	0.33
195	25.71	*unidentified*	-	-	-	0.54	0.14	0.08	0.40	0.44	0.56	1.31	0.15	0.60	0.15	0.51	0.26	0.73
196	25.81	*unidentified*	-	-	-	0.23	0.26	0.20	1.12	0.30	1.38	0.80	0.66	0.82	0.30	1.34	2.29	2.32
197	25.88	β-Bisabolene	1.505	1.505	C15H24	0.34	0.02	0.03	0.20	0.04	0.12	0.06	0.08	0.41	0.04	0.05	0.16	0.16
198	25.94	α-Muurolene	1.490	1.500	C15H24	0.25	0.15	0.55	0.97	0.20	0.63	0.18	0.37	0.84	0.46	0.13	1.43	1.00
199	25.96	β-Curcumene	1.500	1.514	C15H24	0.13	0.11	0.20	0.39	0.10	0.37	0.22	0.18	0.51	0.25	0.22	0.79	0.52
200	26.02	*unidentified*	-	-	-	0.35	0.26	0.52	0.24	0.06	1.19	0.53	0.21	0.40	0.55	0.39	0.84	0.41
201	26.17	*unidentified*	-	-	-	0.09	0.46	0.33	0.76	0.18	1.49	0.35	0.41	0.37	0.48	0.38	01.01	1.18
202	26.22	*unidentified*	-	-	-	0.03	0.06	0.06	0.25	0.15	1.06	0.10	0.09	0.06	0.39	0.07	0.60	0.34
203	26.28	γ-Cadinene	1.510	1.513	C15H24	0.76	0.10	0.57	0.27	0.13	0.23	0.03	0.09	01.03	0.41	1.14	0.62	0.15
204	26.39	*unidentified*	-	-	-	0.77	0.34	0.58	0.21	0.02	0.27	0.29	0.07	0.76	0.34	0.87	0.03	0.06
205	26.47	α-Alaskene	1.521	1.512	C15H24	0.77	0.08	0.08	0.05	0.01	0.09	0.11	0.09	0.01	0.36	0.24	0.04	0.18
206	26.64	δ-Cadinene	1.514	1.522	C15H24	0.13	0.11	0.13	0.39	0.01	0.26	0.28	0.05	0.01	0.30	0.50	0.07	0.01
207	26.66	*unidentified*	-	-	-	0.25	0.17	0.19	1.24	0.01	0.07	0.61	0.07	0.02	0.53	0.76	0.01	0.06
208	26.81	*unidentified*	-	-	-	0.02	0.00	0.00	0.44	0.18	0.45	0.19	0.13	0.00	0.04	0.07	0.37	0.68
209	26.82	*unidentified*	-	-	-	0.12	0.01	0.01	02.03	0.83	1.95	0.70	0.68	0.00	0.20	0.42	1.25	3.30
210	26.94	trans-Calamenene	1.516	1.521	C15H22	0.82	0.11	0.52	0.01	0.02	0.09	2.94	0.89	1.91	1.34	1.13	0.21	0.06
211	26.99	cis-Calamenene	1.535	1.528	C15H22	1.12	0.11	0.62	1.27	0.03	0.04	2.85	0.87	1.91	1.32	03.06	0.03	0.02
212	27.09	*unidentified*	-	-	-	0.41	0.26	0.27	3.59	0.69	1.72	2.65	0.84	0.14	0.73	03.02	0.51	04.01
213	27.09	trans-Cadina-1,4-diene	1.537	1.533	C15H24	0.25	0.00	0.02	0.11	0.04	0.11	0.04	0.04	0.64	0.04	0.01	0.34	0.12
214	27.32	*unidentified*	-	-	-	0.19	0.03	0.07	0.12	0.01	0.01	0.00	0.01	0.58	0.06	0.21	0.15	0.02
215	27.32	α-Cadinene	1.540	1.537	C15H24	0.25	0.03	0.09	0.12	0.01	0.02	0.07	0.00	0.65	0.12	0.33	0.17	0.02
216	27.54	Selina-3,7(11)-diene	1.550	1.545	C15H24	0.22	0.03	0.07	0.23	0.04	0.18	0.13	0.04	0.31	0.09	0.19	0.07	0.10

217	27.55	α-Calacorene	1.554	1.544	C15H20	0.04	0.01	0.02	0.15	0.01	0.02	0.32	0.06	0.51	0.07	0.32	0.02	0.06
218	27.71	α-Calamenene	1.555	1.544	C15H20	0.53	0.05	0.26	0.01	0.00	0.01	0.00	0.00	0.28	0.60	0.56	0.01	0.00
219	27.79	β-Calamenene	1.557	1.564	C15H20	0.61	0.18	0.31	1.16	0.07	0.15	0.23	0.32	0.00	0.70	0.82	0.32	0.30
220	28.08	*unidentified*	-	-	-	0.00	0.01	0.00	0.01	0.01	0.01	0.01	0.00	0.00	0.01	0.01	0.01	0.02
221	28.17	*unidentified*	-	-	-	0.00	0.00	0.00	0.02	0.01	0.01	0.01	0.00	0.00	0.00	0.00	0.01	0.02
222	29.93	*unidentified*	-	-	-	0.01	0.00	0.01	0.18	0.04	0.13	0.23	0.04	0.58	0.04	0.07	0.02	0.42
223	29.94	*unidentified*	-	-	-	0.02	0.01	0.02	0.73	0.08	0.40	0.37	0.03	0.26	0.07	0.15	0.03	0.76
224	30.05	trans-Vettonal	1.557	1.555	C14H22O	0.00	0.00	0.00	0.01	0.09	0.11	0.02	0.06	0.64	0.02	0.03	0.08	0.09
225	31.01	*unidentified*	-	-	-	0.00	0.00	0.00	0.09	0.09	0.02	0.03	0.01	0.43	0.05	0.03	0.02	0.05
226	32.27	*unidentified*	-	-	-	0.00	0.00	0.00	0.03	0.01	0.02	0.01	0.00	0.38	0.00	0.00	0.01	0.02
227	32.28	*unidentified*	-	-	-	0.00	0.00	0.00	0.01	0.01	0.01	0.00	0.00	0.38	0.01	0.00	0.01	0.01
228	32.38	Cadalene	1.686	1.675	C15H18	0.18	0.01	0.10	0.00	0.01	0.00	0.00	0.00	0.12	0.28	0.35	0.00	0.00
229	35.92	*unidentified*	-	-	-	0.57	1.66	0.00	0.01	0.01	0.01	0.01	0.01	0.01	0.00	0.00	0.02	0.02
230	35.97	*unidentified*	-	-	-	1.00	1.35	0.00	0.01	0.01	0.02	0.01	0.01	0.01	0.00	0.00	0.02	0.02
231	36.11	Benzyl benzoate	1.802	1.759	C14H12O2	0.58	0.01	0.21	0.01	0.04	0.01	0.01	0.01	0.36	0.00	0.74	0.03	0.02
% Total						100	100	100	100	100	100	100	100	100	100	100	100	100

* MqJ: *Melipona quadrifasciata* - Jaguariúna / MqCV: *Melipona quadrifasciata* - Cabo Verde / MqM: *Melipona quadrifasciata* - Muzambinho / TaJ: *Tetragonisca angustula* – Jaguariúna / TaM: *Tetragonisca angustula* – Muzambinho / MrCV: *Melipona rufiventris* - Cabo Verde / NtJ: *Nannotrigona testaceicornis* - Jaguariúna / NtM: *Nannotrigona testaceicornis* - Muzambinho / PdCV: *Plebeia droryana* - Cabo Verde / PdJ: *Plebeia droryana* - Jaguariúna / FvJ: *Frieseomelitta varia* – Jaguariúna / MbJ: *Melipona bicolor* – Jaguariúna / MmJ: *Melipona marginata* – Jaguariúna.

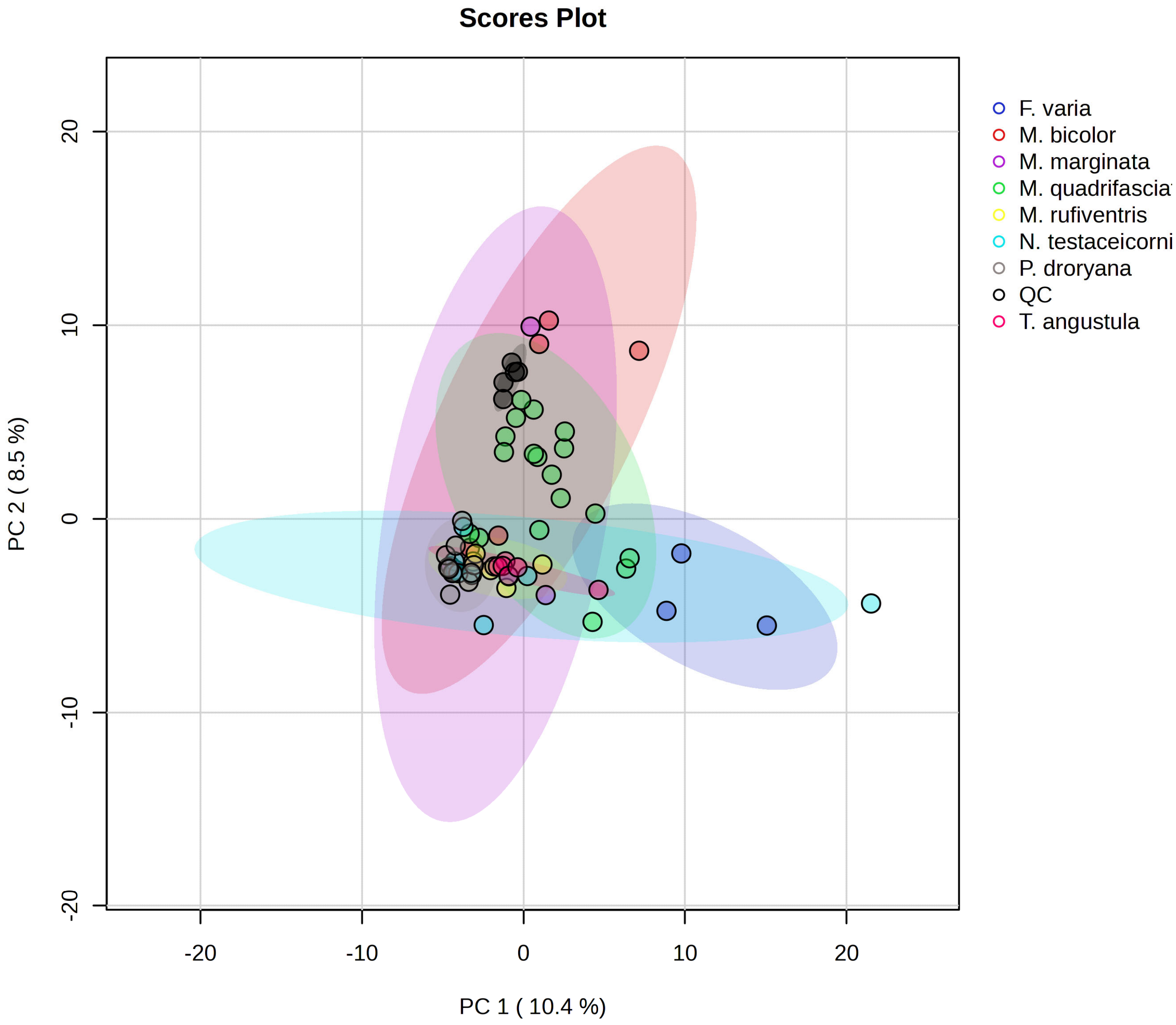

Figure S1. Principal Component Analysis (PCA) was performed using samples collected from the regions of Jaguariúna (J), Campo Verde (CV), Muzambinho (M), and quality control (QC) samples.

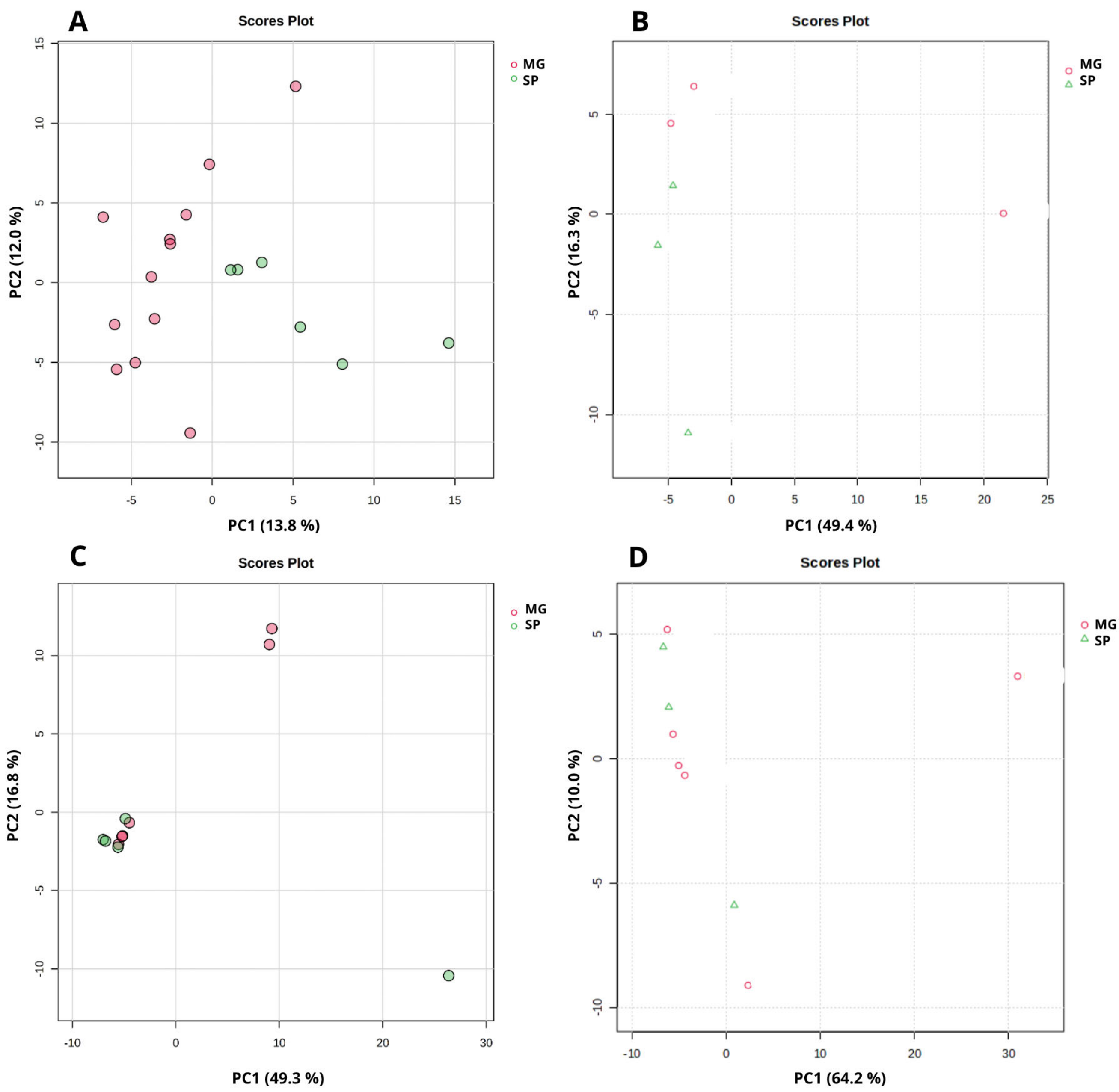

Figure S2. Principal Component Analysis (PCA) based on the chromatographic profiles obtained by GC–MS from (geo)propolis samples collected in Southeastern Brazil (states of São Paulo, SP, and Minas Gerais, MG). (A) *Melipona quadrifasciata*; (B) *Tetragonisca angustula*; (C) *Plebeia droryana*; and (D) *Nannotrigona testaceicornis*. The plots show the distribution of samples according to the first two principal components (PC1 and PC2).

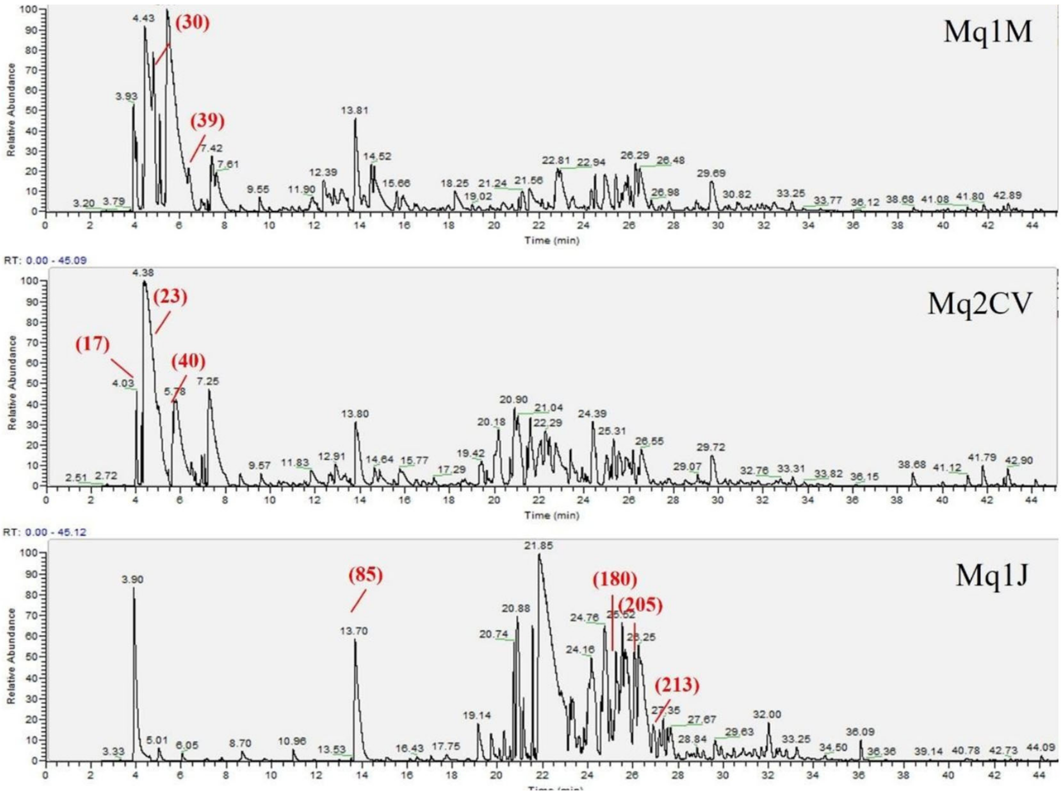

Figure S3. Chromatograms of propolis samples produced by the bee species *M. quadrifasciata* (Mq1M, sample from Muzambinho; Mq2CV, sample from Cabo Verde; and Mq1J, sample from Jaguariúna).

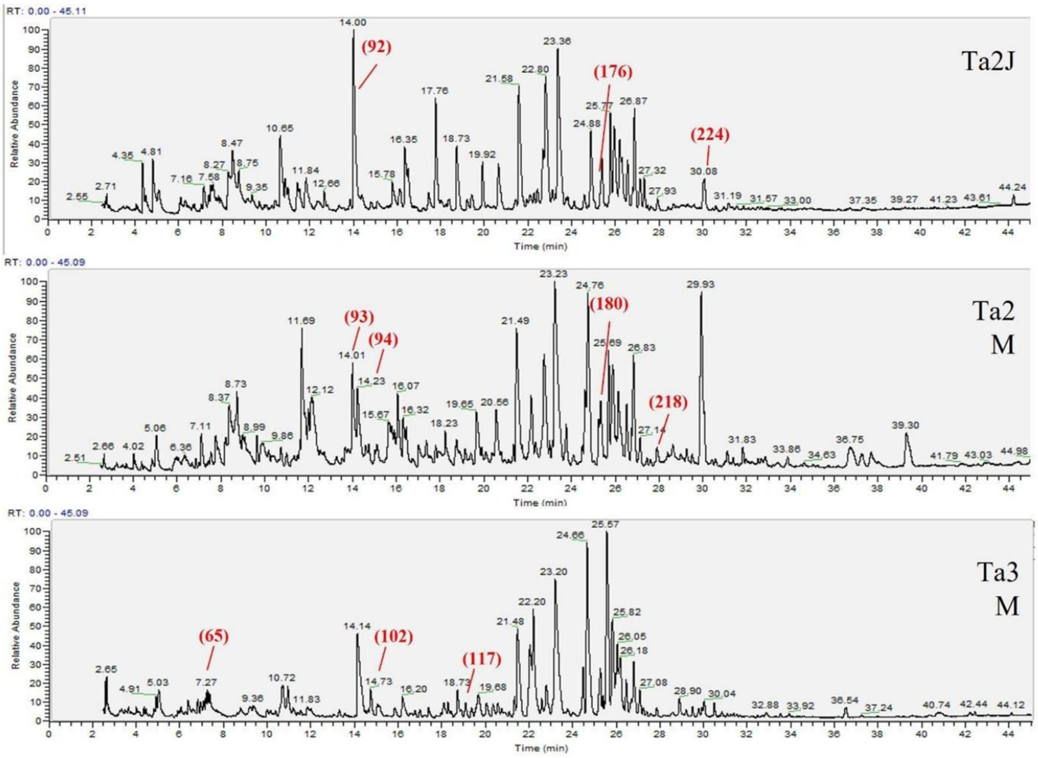

Figure S4. Chromatograms of propolis samples produced by the bee species *T. angustula*, collected in the Brazilian states of São Paulo (Ta2J) and Minas Gerais (Ta2M and Ta3M).

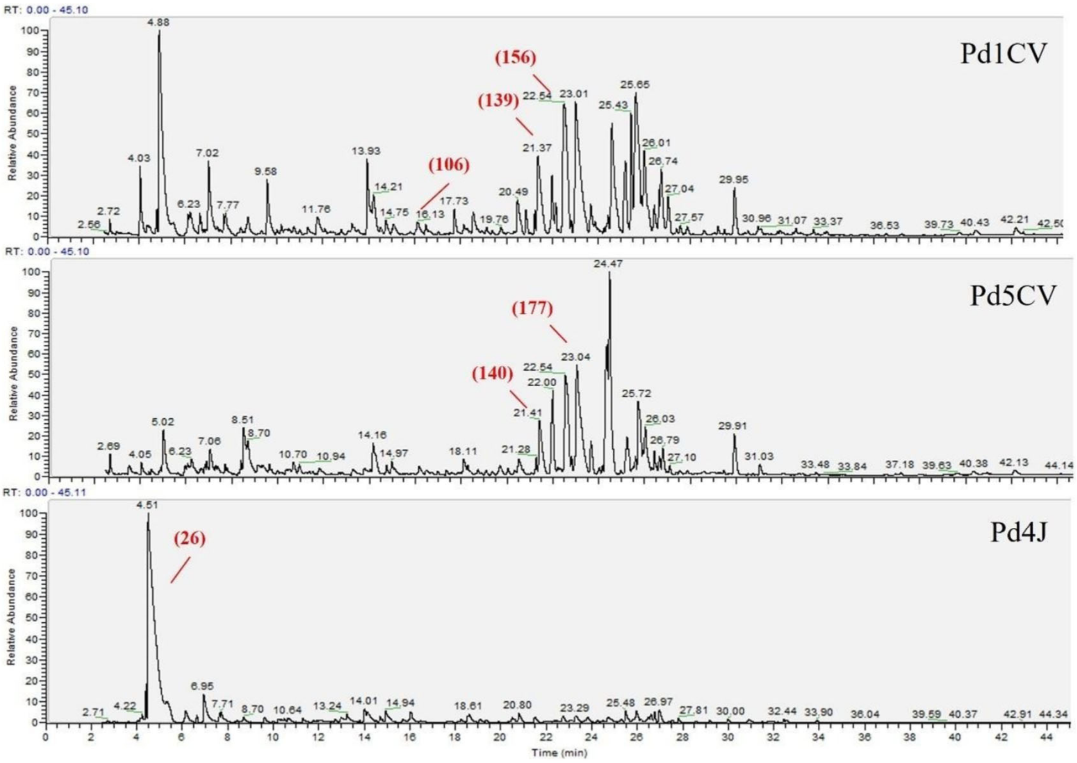

Figure S5. Chromatograms of propolis samples produced by the bee species *P. droryana* (Pd1CV and Pd5CV, collected in the Cabo Verde region, Minas Gerais, MG; and Pd4J, collected in the Jaguariúna region, São Paulo, SP, Brazil).

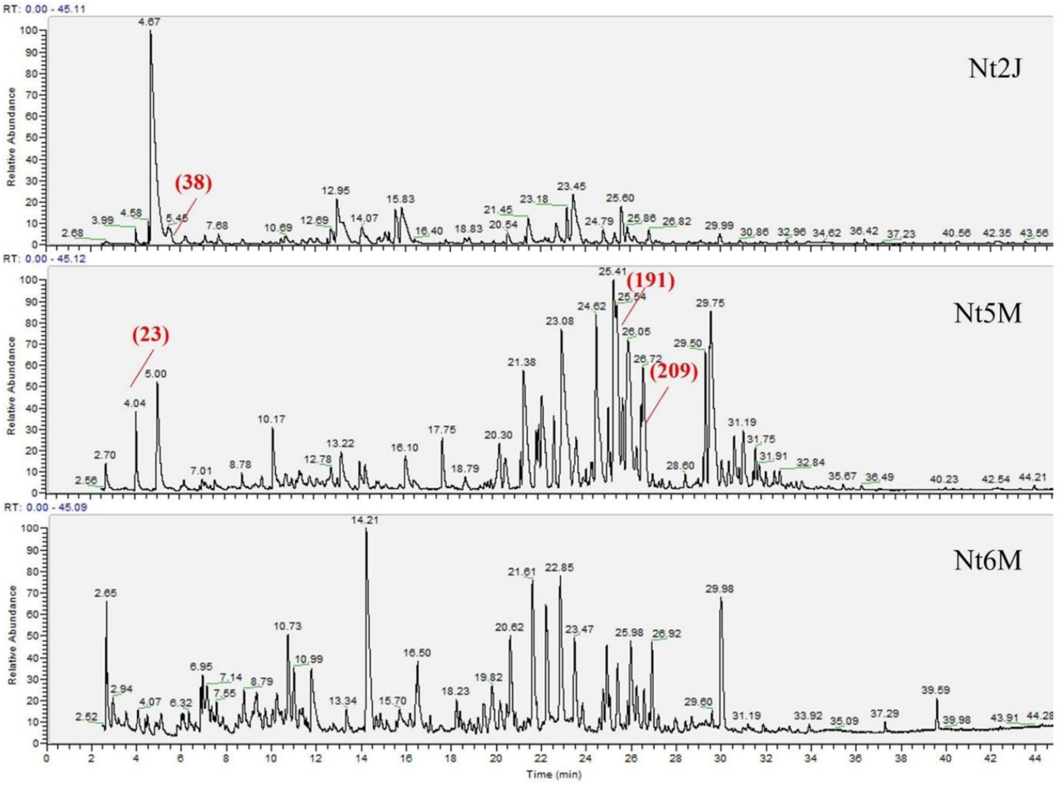

Figure S6. Chromatograms of propolis samples produced by the bee species *N. testaceicornis* (Nt2J, collected in Jaguariúna, São Paulo, SP; and Nt5M and Nt6M, collected in Muzambinho, Minas Gerais, MG, Brazil).
